# QishenYiqi dripping pill protects against myocardial ischemia/reperfusion injury via suppressing excessive autophagy and NLRP3 inflammasome based on network pharmacology and experimental pharmacology

**DOI:** 10.3389/fphar.2022.981206

**Published:** 2022-09-09

**Authors:** Meng Li, Yueyao Wang, Zhongwen Qi, Zhuo Yuan, Shichao Lv, Yawei Zheng, Zhipeng Yan, Mingyang Wang, Huanjie Fu, Xinbiao Fan, Nan Ji, Ming Liu, Zhuyuan Fang

**Affiliations:** ^1^ Institute of Hypertension, Jiangsu Province Hospital of Chinese Medicine, Affiliated Hospital of Nanjing University of Chinese Medicine, Nanjing, China; ^2^ The Second Affiliated Hospital, Guangzhou University of Chinese Medicine, Guangzhou, China; ^3^ Institute of Gerontology, Xiyuan Hospital, China Academy of Chinese Medical Sciences, Beijing, China; ^4^ Department of Psychosomatic Medicine, First Teaching Hospital of Tianjin University of Traditional Chinese Medicine, Tianjin, China; ^5^ Geriatric Department, First Teaching Hospital of Tianjin University of Traditional Chinese Medicine, Tianjin, China; ^6^ Graduate School, Tianjin University of Traditional Chinese Medicine, Tianjin, China; ^7^ School of Basic Medical Sciences, Tianjin Medical University, Tianjin, China

**Keywords:** QSYQ, myocardial ischemia, reperfusion, autophagy, NLRP3 inflammasome

## Abstract

**Background:** Myocardial ischemia/reperfusion (I/R) injury is associated with multiple serious clinical manifestations. Autophagy is upregulated in a short period of ischemia and further enhanced during reperfusion phase, which was considered as a “double-edged sword” in the pathological process of myocardial I/R injury. In addition, NLRP3 inflammasome triggers myocardial inflammatory response, which leads to cardiomyocyte death via pyroptosis and promotes subsequent myocardial remodelling. Qishen Yiqi Dripping Pill (QSYQ) has been recognized as a potential protective agent of cardiovascular diseases.

**Objective:** We predicted the bioactive compounds, targets and pathways of OSYQ intervening on myocardial I/R injury by network pharmacology. Furthermore, we investigated the effect of QSYQ on myocardial I/R injury and explored its underlying mechanism via autophagy and NLRP3 Inflammasome.

**Methods:** Bioactive compounds, targets of QSYQ and relevant targets of myocardial I/R injury were collected from public databases. The protein-protein interaction network, Gene ontology and KEGG pathway enrichment analysis were carried out to screen the key compounds, target genes, functional annotation and pivotal pathways. Molecular docking was used to validate the binding association between target genes and key bioactive ingredients. Furthermore, sixty SD rats were randomized into four groups: 1) sham, 2) model, 3) captopril and 4) QSYQ pretreatment (14 days before and after surgery). Each arm was subjected to ischemia/reperfusion surgery except sham arm (30 min coronary ligation, then reperfusion). Left ventricular (LV) function were evaluated and the hearts were used to evaluate size of myocardial infarction, cardiomyocyte fibrosis, and myocardial autophagosomes.

**Results:** The network pharmacology revealed the mechanism of QSYQ intervening on myocardial I/R injury might be related to NOD-like receptor signaling pathway, PI3K-Akt signaling pathway, autophagy-animal, etc., Molecular-docking suggested the core target proteins had good binding association with bioactive compounds of QSYQ. The experiment confirmed that QSYQ attenuated myocardial infarct size, decreased inflammatory infiltration and collagen fiber deposition and alleviated the autophagosome and myocardium ultrastructure injury, leading to LV systolic function improvement. The possible mechanism of cardioprotection was due to regulating autophagy-related proteins, activating PI3K/Akt-mTOR signaling pathway, and inhibiting activation and assembly of NLRP3 inflammasome.

**Conclusion:** QSYQ ameliorated myocardial I/R injury via suppressing excessive autophagy and NLRP3 Inflammasome.

## 1 Introduction

Acute myocardial infarction (AMI) is the principal cause of morbidity and mortality throughout the world. Reperfusion strategies remain the current standard treatment for AMI. To date, successful and timely myocardial reperfusion with primary percutaneous coronary intervention or thrombolytic agents is the most effective strategy to reduce the area of myocardial infarction and improve prognosis (Reed et al., 2017). However, recent studies have demonstrated that reperfusion has the potential to induce subsequent paradoxical myocardial injury and exacerbate cardiac dysfunction, a phenomenon known as myocardial ischemia/reperfusion (I/R) injury (Heusch, 2020). A variety of pathological processes are involved in I/R injury including autophagy, apoptosis, pyroptosis, immune activation, mitochondrial injury, inflammatory reaction, and oxidative stress (Wu et al., 2018). However, there are still no effective therapy for preventing and treating I/R injury.

It is well known that autophagy is an intracellular catabolic process that plays an important role in the degradation and recycling of long-lived or excess proteins and senescent organelles to clear away damaged and dysfunctional cellular components and maintain homeostasis (Cao et al., 2021). However, emerging evidence suggests that autophagy is also essential for cell death in response to some types of stress. Current studies indicate that autophagy is upregulated in a short period of ischemia and further enhanced during reperfusion phase, which can be a “double-edged sword” in pathological process of I/R injury (Mokhtari and Badalzadeh, 2021). During a short period of ischemia, ATP production is reduced due to impaired mitochondrial function and phosphorylation uncoupling. Increased AMP/ATP ratio initiates cardiomyocyte autophagy. Free fatty acids and amino acids are released during autophagy process, and ATP is generated through the tricarboxylic acid cycle to compensate for the energy crisis in myocardial ischemia. Autophagy is an energy-recovering process during ischemia, which is crucial to the survival of cardiomyocyte and has protective effects (Aghaei et al., 2019). However, reperfusion can further enhance autophagy, and uncontrolled excessive autophagy can purge necessary proteins and organelles, which leads to autophagic cardiomyocyte death and further aggravating myocardial injury (Lin et al., 2018).

The Nod-like Receptor Protein-3 (NLRP3) inflammasome is a multiprotein complex consisting of a sensor (NLRP3), an adaptor (ASC, also termed PYCARD), and an effector (caspase-1) (He et al., 2016). NLRP3 is an intracellular sensor that detects environmental stimulus and endogenous danger signals, which leads to formation and activation of NLRP3 inflammasome. The NLRP3 inflammasome assembly induces release of caspase-1-dependent pro-inflammatory cytokines IL-1β and IL-18, and gasdermin D-regulated pyroptosis (Huang et al., 2021a). Emerging evidence indicates that NLRP3 inflammasome plays an essential role in the process of I/R injury, which triggers myocardial inflammatory response, leads to cardiomyocyte death by pyroptosis, and promotes subsequent myocardial remodelling (Guo et al., 2016). Glycogen synthase kinase-3 (GSK-3β) is an important mediator of inflammatory response, and GSK-3β activation is associated with multiple cardiovascular diseases (Wang et al., 2020). Increasing evidence suggests that GSK-3β acts as a molecular determinant of spatio-temporal dynamics of NLRP3 inflammasome activation. NLRP3 stimuli initiates GSK3-β activation with subsequent binding to NLRP3 that facilitates NLRP3 recruitment to mitochondria and transition to Golgi network where it is retained for inflammasome assembly. GSK3-β activation also promotes sustained NLRP3 oligomerization (Arumugam et al., 2022).

Qishen Yiqi Dripping Pill (QSYQ) is a standardized Chinese herbal preparation approved by China Food and Drug Administration (CFDA) (Approval Number of CFDA: Z20030139) in 2003 for cardiovascular diseases treatment (Zhang et al., 2012). QSYQ consists of Hedysarum Multijugum Maxim, Radix Salviae, Panax Notoginseng, and Dalbergia Odorifera with a proportion of 10:5:1:0.067 (Huang et al., 2021b). Currently, a randomized controlled trial has suggested that QSYQ can improve ventricular remodeling and function, and improve symptoms and prognosis in patients with chronic heart failure (Chang et al., 2019). A pilot randomized study has indicated that QSYQ can reduce myocardial injury and preserve microvascular function in patients undergoing selective percutaneous coronary intervention (He et al., 2018). Moreover, an increasing number of experimental studies have indicated that QSYQ can alleviate myocardial I/R injury by improving cardiac function, inhibiting ventricular remodeling and myocardial fibrosis, reducing inflammation, regulating energy metabolism, and ameliorating myocardial microcirculatory disturbance (Chen et al., 2015; Han et al., 2019). QSYQ also can attenuate cardiac hypertrophy and myocardial fibrosis induced by pressure overload, and regulate myocardial collagen metabolism in experimental autoimmune myocarditis (Lv et al., 2021). In the current study, we used network pharmacology to predict the bioactive compounds, targets and pathways of QSYQ intervening on myocardial I/R injury, and performed molecular docking to validate the binding association between the target genes and the critical bioactive ingredients. Furthermore, we investigated the effect of QSYQ on myocardial I/R injury and explored its mechanism of action by autophagy and NLRP3 Inflammasome.

## 2 Materials and methods

### 2.1 Collection of metabolite targets of Qishen Yiqi dripping pill effective components

The main composition of QSYQ: Huangqi (Hedysarum Multijugum Maxim, HM), Danshen (Radix Salviae, RS), Sanqi (Panax Notoginseng, PN), Jiangxiang (Dalbergiae Odoriferae Lignum, DO). The metabolites of active components in all herbal compounds of QSYQ were collected from YaTCM (http://cadd.pharmacy.nankai.edu.cn/yatcm/home) and TCMIO (http://tcmio.xielab.net), and the screening criteria were oral bioavailability (OB) ≥30% and drug-likeness (DL) ≥0.18. The metabolite targets were identified from ChEMBL database (https://www.ebi.ac.uk/chembl/) and TCMIO. For drug screening and evaluation, comprehensive information on all herbal ingredients obtained from distinct databases was used.

### 2.2 Acquisition of therapeutic targets for myocardial reperfusion injury

Potential targets associated with myocardial I/R injury were screened using “myocardial reperfusion injury” as the keyword from the TTD (http://db.idrblab.net/ttd/) and DisgenetGene database (http://www.disgenet.org). Furthermore, compound names were standardized according to PubChem CIDs (https://pubchem.ncbi.nlm.nih.gov/). The obtained targets were submitted to the UniProt website (http://www.uniprot.org) to verify their genetic names.

### 2.3 Gene ontology and KEGG pathway enrichment analysis

Gene Ontology (GO) enrichment analysis for overlapping genes was performed by the GO (http://www.geneontology.org/) to identify biological processes. Target proteins were classified and displayed by Biological Process (BP), Cellular Component (CC) and Molecular Function (MF). The KEGG pathway database (www.kegg.jp/kegg/pathway.html) was used for KEGG pathway enrichment analysis, and the species were setted as “*Homo sapiens*” to systematically explore pathways related to the shared targets.

### 2.4 Construction of protein-protein interaction network and metabolite-target-pathway network

The protein interaction information of protein-protein interaction (PPI) network was constructed using STRING database (https://string-db.org/) with the species limited as “*Homo sapiens*”. R-studio network was utilized for visualization. R-studio network was used to construct the metabolin-target-pathway network topology.

### 2.5 Molecular docking to active component metabolites

The 3D structure of the compounds were obtained from PubChem database (https://pubchem.ncbi.nlm.nih.gov/) and TCMSP database (http://tcmspw.com/tcmsp.php), and the structure of target proteins were obtained from RCSB PDB (https://www.rcsb.org/). AutoDock Tools 1.5.6 was applied to predict the binding energy of compounds and proteins. The docking results of the observed compounds and proteins were analyzed by using PyMOL software. Finally, PyMOL software was used to visualize the docking results and establish the docking interaction mode.

### 2.6 Animals

Male Sprague-Dawley (SD) rats aged 5–6 weeks, SPF grade, weight 200 ± 20 g were purchased from Beijing Huafukang Bioscience Co., Ltd. (certificate No. SCXK Beijing 2016-0002). Animals were kept under standard conditions with an average temperature of 22°C ± 2°C, an average relative humidity of 55% ± 10% and a defined circadian rhythm of 12 h of light and 12 h of darkness. All animal procedures performed in this study were approved by the Animal Ethics Committee of Tianjin University of Traditional Chinese Medicine (No. TCM-LAEC2018033), China.

### 2.7 Materials

All reagents used in the study were as follows: QSYQ pill (Tasly Pharmaceutical CO. Ltd., Tianjin, China); captopril tablets (Changzhou Pharmaceutical Factory Co., Ltd. Changzhou, China); hematoxylin-eosin stain kit (Tianjin Baihao Biotechnology Co., Ltd. Tianjin, China); masson trichrome staining kit, RIPA lysate, protease inhibitor, and phosphatase inhibitor (Leagene Biotechnology Co., Ltd. Beijing, China); TTC Stain Kit (Nanjing Jiancheng Bioengineering Institute Co., Ltd. Nanjing, China); WGA (Sigma, United States); BCA protein concentration test kit (Boster Biological Technology Co., Ltd. Wuhan, China); Goat anti-Rabbit IgG (H + L) Cross-Adsorbed Secondary Antibody (Thermo Fisher Scientific, United States); Cyanine3 and Cyanine5 (Biolite Biotech, Inc., Xi’an, China); CoraLite488-conjugated Affinipure Goat Anti-Rabbit IgG (H + L), HRP-conjugated Affinipure Goat Anti-Mouse IgG, HRP-conjugated Affinipure Goat Anti-Rabbit IgG, ECL chemiluminescence detection kit, p53 Antibody and GSK-3β Antibody (Proteintech Group, Inc., United States); Akt Antibody, Phospho-Akt (Ser473) Antibody, mTOR Antibody, Phospho-mTOR (Ser2448) Antibody, and Beclin1 Antibody (Cell Signaling Technology, United States); Anti-LC3B antibody [EPR18709]-Autophagosome Marker, Anti-NLRP3 antibody, and Mouse monoclonal anti-GAPDH (Abcam, United States); ASC Antibody, ATG5 Antibody, Cleaved-IL-1β (Asp116) Antibody, PI3K p85/p55 Antibody, Phospho-PI3K p85 (Tyr458)[Tyr467]/p55 (Tyr199) Antibody, Bcl-2 Antibody, Gasdermin D Antibody, and Cleaved-Caspase-1 (Asp296), p20 Antibody, and Caspase-1 Antibody (Affinity Biosciences, Liyang, China).

### 2.8 Establishment of myocardial I/R injury model and experimental protocol

SD rats were randomized into sham group, model group, captopril group and QSYQ group. We followed the methods of Song XD et al. to establish the myocardial I/R injury model (Song et al., 2019). SD rats were anaesthetized by intraperitoneal injection of 1% pentobarbital sodium (40 mg/kg), and mechanically ventilated by animal ventilator after endotracheal intubation. A 5/0 noninvasive suture needle was inserted 2 mm below the left auricular appendage to make a slipknot around the left anterior descending artery (LAD) and induce myocardial ischemia. After surgically ligating the coronary artery, the myocardium below the ligation site turned gray or cyanosis, and the electrocardiogram (ECG) showed ST-segment elevation, suggesting the experimental model was successful. After 30 min of ischemia, the slipknot was released and the myocardium received reperfusion. During myocardial reperfusion, tissue hyperemia occurred in the local myocardium, and the elevated ST-segment depressed by more than 1/2, which indicated a successful reperfusion. The rats in the sham group underwent the same operation but without occluding the suture under LAD. The ECG changes were recorded with MouseMonitor^TM^S (INDUS) real-time small animal vital signs monitor.

Angiotensin converting enzyme inhibitors (ACEI) can improve myocardial remodeling, prevent heart failure, and significantly reduce the mortality and the risk of recurrent cardiovascular events in patients with coronary heart disease (van-Vark et al., 2012; Ferrari and Rosano, 2013; Cheng et al., 2014). ACEI significantly reduce the incidence of adverse cardiovascular events and improve clinical prognosis in patients with coronary heart disease undergoing coronary revascularization (Mohr et al., 2013). Several international guidelines have recommend ACEI for patients with acute coronary syndrome (ACS) without contraindications (Snow et al., 2004). Therefore, we chose captopril as a positive control drug. Equivalent dosage of captopril and QSYQ were calculated according to the body surface area (Xu, 2002). The captopril group and QSYQ group were prophylactically administered captopril with 9 mg/kg/d and QSYQ with 270 mg/kg/d, respectively, by gavage 14 days before model establishment, and until 14 days after model establishment. The sham group and model group were given equal volume of distilled water by gavage 14 days before model establishment, and until 14 days after model establishment. The myocardial tissue specimens were harvested 14 days after model establishment.

### 2.9 Assessment of cardiac function via echocardiography

Echocardiography was performed 14 days after model establishment. 95% oxygen + 5% isoflurane were used in the anesthesia induction box, and 97%–98% oxygen + 2%–3% isoflurane were used for continuous anesthesia during surgery. We followed the methods of Lv SC et al. to assess the cardiac function (Lv et al., 2020). Each rat was fixed on the operating table, the skin of the chest was extensively scraped for skin preparation, and then the chest was applied with coupling agent. A long-axis section image of parasternal left ventricle was obtained by using Vevo^®^ 2100 ultra-high resolution small animal ultrasound imaging system (probe MS-250, frequency 21 MHz) under two-dimensional mode (B-Mode). The sampling line was placed at the maximum diameter of the left ventricle to display the M-type ultrasound image and measured using a long-axis measurement package (PLAX). Left ventricular end diastolic diameter (LVIDd), left ventricular end systolic diameter (LVIDs), left ventricular ejection fraction (LVEF), and left ventricular fraction shortening (LVFS) were detected. Each index was measured for three cardiac cycles to calculate the mean value.

### 2.10 Observation of myocardial tissue pathological morphology by pathological staining

Measurement of myocardial infarct size by triphenyltetrazolium chloride (TTC) staining: the myocardial tissue was frozen at −20°C for 20–30 min, and cut into slices of 2–3 mm thickness that was parallel to the coronary sulcus and below the heart ligature. The slices were incubated in TTC incubation solution at 37°C for 30 min away from light. Then observed and analyze the myocardial infarction area (white or pale) and non-infarction area (red). Image analysis software (Image-Pro plus Version 6.0) was used to calculate the proportion of infarct myocardium to the entire myocardial tissue. Infarct size (%) = (infarct area/whole heart area) × 100%.

The myocardial tissues were fixed with 4% paraformaldehyde, and conventional ethanol was used for gradient dehydration, transparency, paraffin embedding and continuous sections (5 μm-thick). The myocardium sections were stained with hematoxylin and eosin (H&E) according to HE staining method, and then observed the pathological changes under a light microscope.

The extent of fibrosis was evaluated using Masson trichrome staining. Myocardium sections were stained with Masson trichrome according to Masson staining method. Then five microscopic fields were selected randomly from each section and ImageJ software was used to calculate collagen volume fraction (CVF, CVF = myocardial collagen fiber area/total image area).

To determine the cross-sectional area of the myocardium, wheat germ agglutinin (WGA) staining was used. The myocardial tissues sections were incubated with WGA Alexa Fluor 488 for 1 h. After washing with PBS, the DAPI solution was added to the sections and incubated for 10 min. Observed and collected images in darkroom by using fluorescence microscope, and then assessed the cardiomyocyte cross-sectional area by using ImageJ software.

### 2.11 Observation of myocardial autophagosomes by transmission electron microscope

The myocardial tissues smaller than 1 mm^3^ were fixed with 2.5% glutaraldehyde solution and 1% osmium tetroxide, and the blocks were dehydrated with gradient ethanol and embedded at 35°C for 12 h. Then the myocardial tissues were cut into about 75–80 nm-thick sections. Next, the sections were prepared by double staining with uranylacetate and lead citrate. Finally, the myocardial autophagosomes and its ultrastructure were observed under a transmission electron microscope.

### 2.12 Detection of the critical proteins expression by immunofluorescence staining

Serial sections (5 μm) of myocardial tissue were incubated with anti-LC3B antibody, anti-NLRP3 antibody, anti-ASC antibody, and Caspase-1 antibody overnight at 4°C. Then the sections were incubated with fluorescentdye-conjugated secondary antibodies (Goat anti-Rabbit IgG (H + L) Cross-Adsorbed Secondary Antibody, Cyanine3, Cyanine5 or CoraLite488-conjugated Affinipure Goat Anti-Rabbit IgG (H + L)), and nuclei were counterstained with DAPI. Images were observed by fluorescent microscope and ImageJ software was used for semi-quantitative analysis.

### 2.13 Detection of the target proteins expression by Western blotting

The myocardial tissue samples were added into protein lysis buffer and crushed by ultrasonic comminution instrument. After lysing for 30 min on ice, the protein concentration in the supernatants of tissue lysates was measured by BCA protein assay kit. The protein was separated using 8% sodium dodecyl sulphate polyacrylamide gel electrophoresis (SDS-PAGE) and transferred to polyvinylidene fluoride (PVDF) membranes. The PVDF membrane was incubated for 2 h in 5% skim milk powder. Primary antibodies including Akt antibody (dilution 1:1,000), phospho-Akt (Ser473) antibody (dilution 1:1,000), PI3K p85/p55 antibody (dilution 1:1,500), phospho-PI3K p85 (Tyr458)[Tyr467]/p55 (Tyr199) antibody (dilution 1:1,500), mTOR antibody (dilution 1:1,000), phospho-mTOR (Ser2448) antibody (dilution 1:1,000), LC3B antibody (dilution 1:800), Beclin1 antibody (dilution 1:1,000), Bcl-2 antibody (dilution 1:1,000), p53 antibody (dilution 1:4,000), GSK-3β antibody (dilution 1:1,000), NLRP3 antibody (dilution 1:1,000), ASC antibody (dilution 1:1,000), Cleaved-Caspase-1 (Asp296) p20 antibody (dilution 1:500), ATG5 antibody (dilution 1:1,000), Cleaved-IL-1β (Asp116) antibody (dilution 1:800), Gasdermin D (dilution 1:1,000) and anti-GAPDH (dilution 1:1,000) were added and incubated at 4°C overnight. Then, the membranes were incubated with appropriate horseradish peroxidase (HRP)-conjugated secondary antibody (dilution 1:4,000) for an hour at room temperature. ECL luminescent reagent was used to visualize the bands, and exposure imaging method was used to perform the automatic gel imaging system. Image Lab software was used to detect the expression of protein bands.

### 2.14 Statistical analysis

All parameters were expressed as mean ± S.D. Statistical analysis was performed using one-way ANOVA followed by the least significant difference (LSD) test for multiple comparisons. SPSS statistical software (version 11.5, SPSS Inc., Chicago, IL, United States) was used for all statistical analyses. The level of significance was set at *p* < 0.05.

## 3 Results

### 3.1 Metabolite and Potential Targets of Qishen Yiqi dripping pill

After the preliminary pharmacokinetic screening, a total of 111 metabolite components (20 in Huangqi, 65 in Danshen, 1 in Sanqi, and 33 in Jiangxiang), and 477 targets were obtained (Supplementary Table S1).

### 3.2 Myocardial I/R injury-related targets

By searching the TTD and DisgenetGene databases and deleting duplicate values, a total of 178 myocardial I/R injury-related targets were identified (Supplementary Table S2).

### 3.3 Gene ontology and KEGG pathway enrichment analysis

**FIGURE 1:**
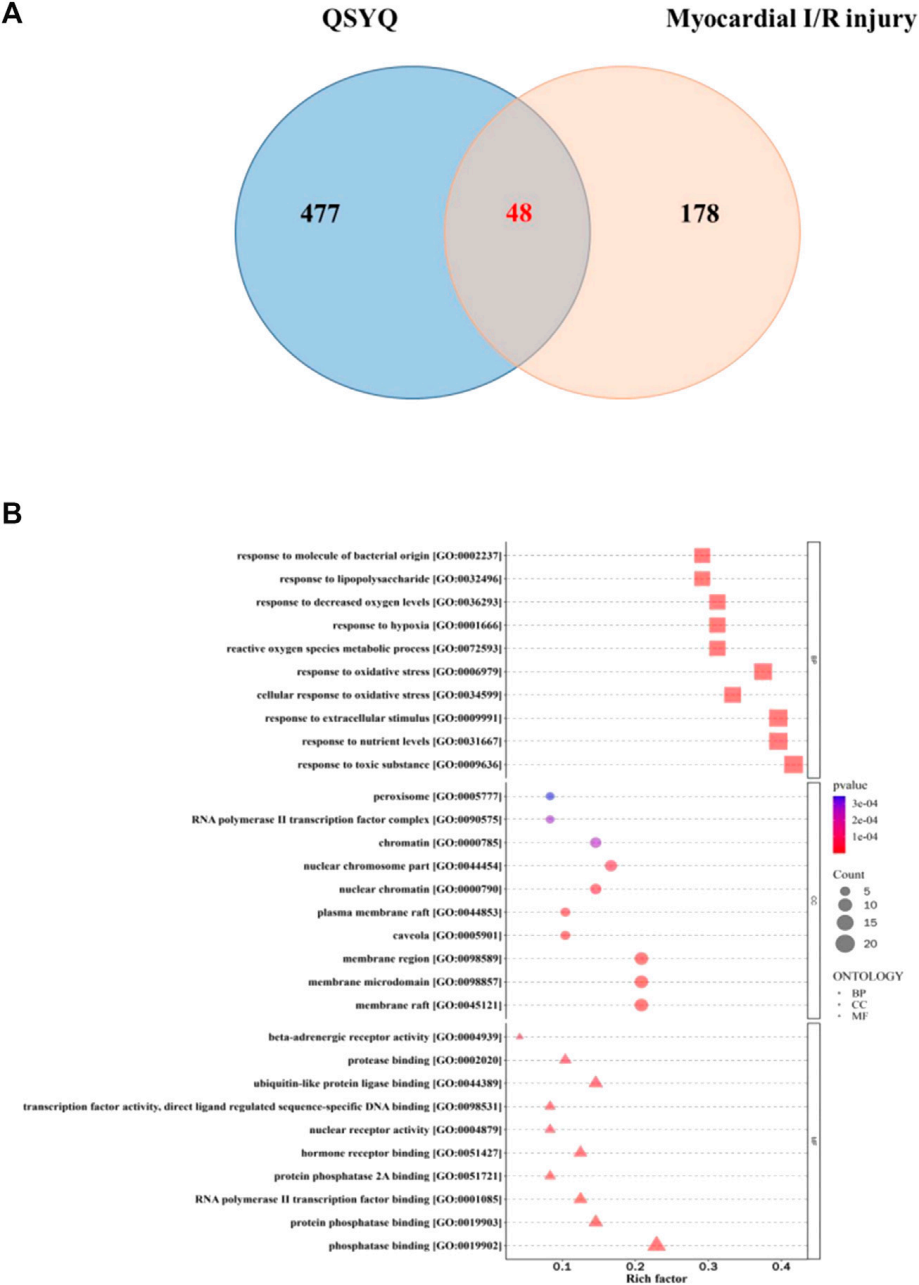
Functional analysis of QSYQ against myocardial I/R injury. **(A)**: Matching map of QSYQ-targeted genes and myocardial I/R injury-targetd genes; **(B)**: GO functional enrichment analysis: the biological processes, cellular component, and molecular function terms were distributed in the ordinate and the abscissa represented rich factor.

A total of 48 overlapping targets between the targets of QSYQ and myocardial I/R injury-related targets were recognized as the therapeutic targets of QSYQ in myocardial I/R injury treatment (Figure 1A). The above 48 targets were analyzed by GO function analysis and KEGG pathway enrichment analyses, and screened with *p <* 0.05. A total of 2087 GO items were identified, among which 1903 items were identified in biological process, including response to molecule of bacterial origin, response to lipopolysaccharide, response to decreased oxygen levels, response to hypoxia, reactive oxygen species metabolic process, etc., A total of 37 entries were for cellular components, including peroxisome, RNA polymerase Ⅱ transcription factor complex, chromatin, nuclear chromosome part, nuclear chromatin, etc., A total of 147 entries were for molecular functions, including beta-adrenergic receptor activity, protease binding, ubiquitin-like protein ligase binding, transcription factor activity, direct ligand regulated sequence-specific DNA binding, nuclear receptor activity, etc., (Figure 1B).

**FIGURE 2:**
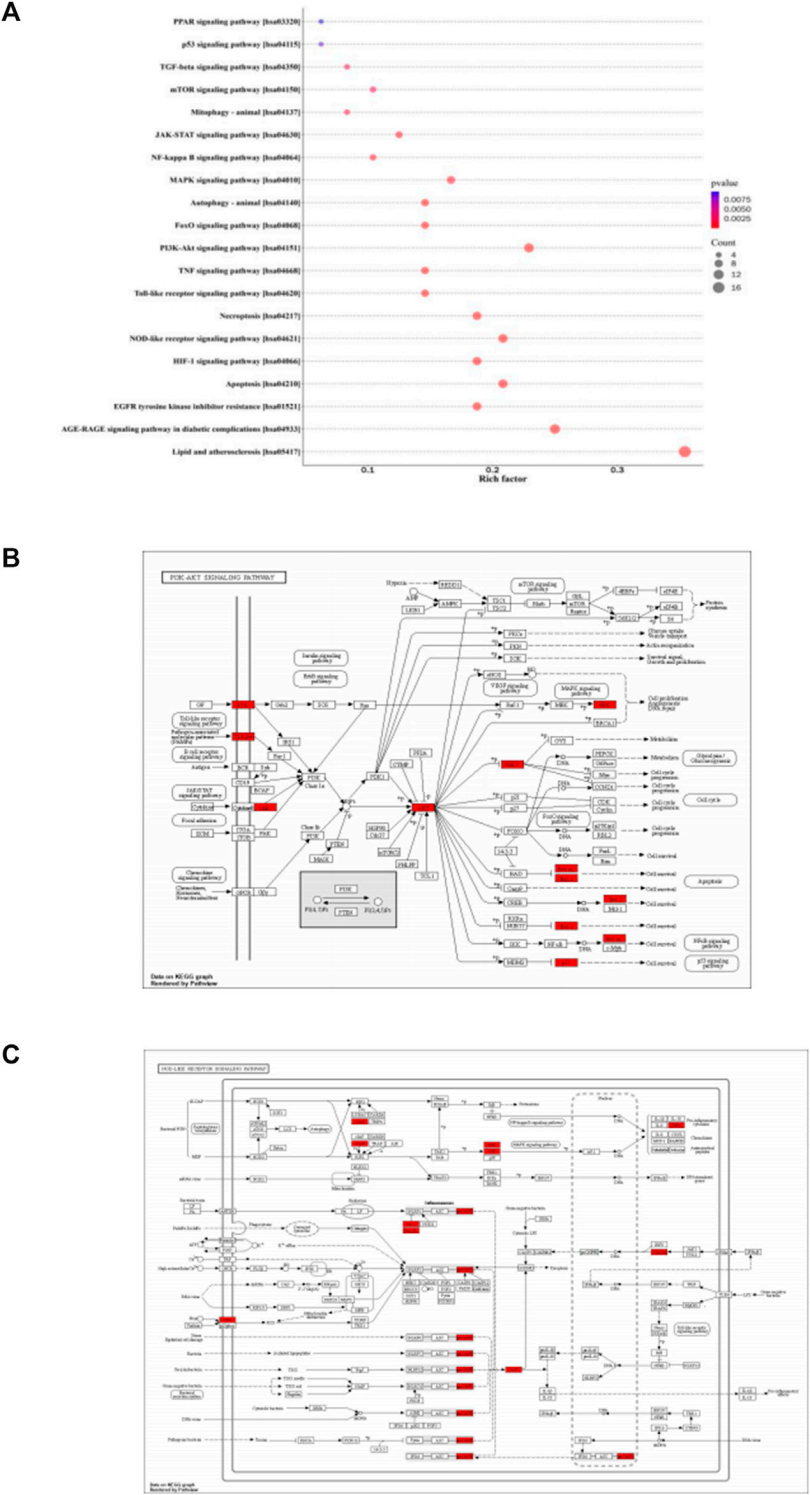
KEGG pathway enrichment analysis. **(A)**: The color of circles represented the *p* values, and the size of circles represented the count. **(B)**: The target proteins were marked bright red on the KEGG pathway map, PI3K-Akt signaling pathway. **(C)**: The target proteins were marked bright red on the KEGG pathway map, NOD-like receptor signaling pathway.

Based on the KEGG pathway enrichment analysis, 155 items were identified, including lipid and atherosclerosis, apoptosis, NOD-like receptor signaling pathway, PI3K-Akt signaling pathway, autophagy-animal, mitophagy-animal, mTOR signaling pathway, p53 signaling pathway, etc., (Figure 2A). The target proteins were marked bright red on the KEGG pathway map, including PI3K-Akt signaling pathway (Figure 2B) and NOD-like receptor signaling pathway (Figure 2C).

### 3.4 PPI network analysis

**FIGURE 3:**
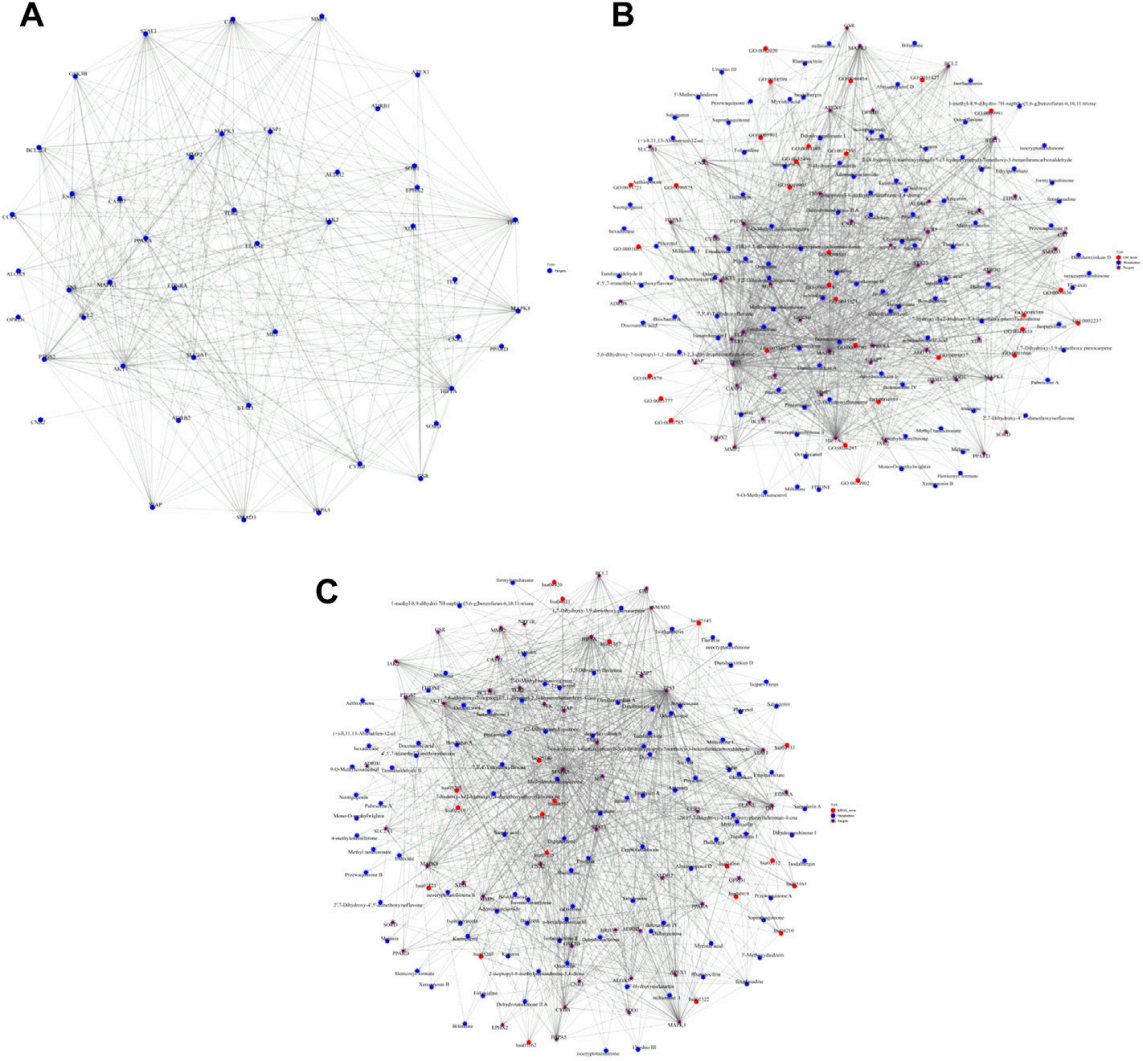
PPI network construction, metabolite-target-GO and metabolite-target-KEGG pathway interaction networks. **(A)**: PPI network comprised 46 nodes and 369 edges. The nodes suggested targets, the edges indicated the interactions between the nodes, and the size of the nodes represented the value of the degree. The key targets of QSYQ in the treatment of myocardial I/R injury included AKT1, Bcl-2, Caspase-1, GSK-3β, p53, etc., **(B)**: Metabolite-target-GO interaction network; **(C)**: Metabolite-target-KEGG pathway interaction network.

The common targets of main active components and myocardial I/R injury were put into STRING database to obtain PPI network, and the mechanism of QSYQ’s action on myocardial I/R injury was intuitively understood. The PPI network comprised 46 nodes and 369 edges. The key targets of QSYQ in the treatment of myocardial I/R injury included AKT1, Bcl-2, Caspase-1, GSK-3β, p53, etc (Figure 3A).

### 3.5 Metabolite-target-GO and metabolite-target-KEGG pathway interaction networks

GO and KEGG pathway analysis were made on the screened active components and targets. The metabolite-target-GO interaction network was shown in Figure 3B. The metabolite-target-KEGG pathway interaction network was shown in Figure 3C.

### 3.6 Molecular docking

**FIGURE 4:**
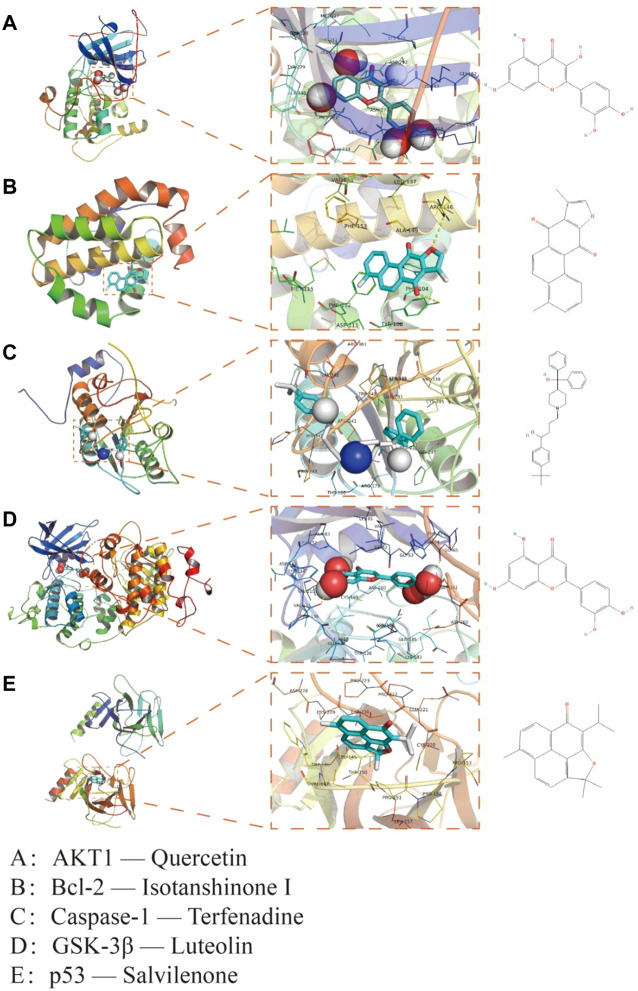
Interaction mode between QSYQ active molecule and core targeted proteins obtained by molecular docking technique. **(A)**: AKT1—Quercetin; **(B):** Bcl-2—Isotanshinone Ⅰ; **(C):** Caspase-1—Terfenadine; **(D):** GSK-3β—Luteolin; **(E):** p53—Salvilenone.

Five targets (AKT1, Bcl-2, Caspase-1, GSK-3β and p53) were selected according to the results of PPI network as the core targets of QSYQ treating myocardial I/R injury. Molecular docking aims to simulate the docking between small molecule ligands and large molecule proteins, and the docking results are evaluated by binding energy (affinity). The affinity less than −7.0 kcal mol^−1^ indicates good binding activity. Lower binding energy indicates better molecular-docking binding effect. Through docking simulations, the docking results of QSYQ active molecule and core targeted proteins were obtained (Table 1). The molecular-docking results suggested that the core targeted proteins (AKT1, Bcl-2, Caspase-1, GSK-3β and p53) had good inter binding with bioactive components of QSYQ. Detailed information about the corresponding active compounds with the best affinity for the core target proteins were shown in Figure 4.

**TABLE 1:** Molecular docking. Through docking simulations, the docking results of QSYQ active molecule and core targeted proteins (AKT1, Bcl-2, Caspase-1, GSK-3β and p53) were obtained. Lower binding energy indicates better molecular-docking binding effect.

Target	ID	Compound Name	Binding Energy (kcal/mol)
AKT1	32043	Quercetin	-8.2
	32358	Apigenin	-7.9
Bcl2	28441	Isotanshinone I	-7.5
	20477	Dalbergin	-6.3
	32053	Luteolin	-6.2
	18414	Physcion	-6.1
	20474	3'-Hydroxymelanettin	-6.1
Caspase-1	2340	terfenadine	-7.7
	2302	fexofenadine	-7.4
	28212	Flavaxin	-6.3
	14476	fluoxetine	-6.1
GSK-3β	32053	Luteolin	-9.5
	32043	Quercetin	-9.4
	32358	Apigenin	-9.3
p53	4549	Salvilenone	-9
	9489	Cryptotanshinone	-8.9
	9496	isocryptotanshinone	-8.9
	8261	neocryptotanshinone ii	-8.9
	28442	Isotanshinone II	-8.7
	28373	Tanshinone I	-8.6
	4656	Miltirone	-8.6
	6257	neocryptotanshinone	-8.6
	4655	Dehydromiltirone	-8.6
	4523	Danshenxinkun B	-8.6
	27968	Melanin	-8.6
	28377	Dehydrotanshinone II A	-8.5
	28328	Przewaquinone B	-8.5
	28441	Isotanshinone I	-8.5
	6855	Przewaquinone A	-8.4
	21774	9-O-Methylcoumestrol	-8.4
	28388	Methylenetanshinquinone	-8.3
	9484	Dihydrotanshinone I	-8.3
	32020	Eriodictyol	-8.2
	32043	Quercetin	-8.2
	28375	Tanshinol A	-8.2
	28374	formyltanshinone	-8.2
	4531	dan-shexinkum b	-8.2
	28446	Tanshilactone	-8.2
	4654	2-isopropyl-8-methylphenanthrene-3,4-dione	-8.1
	32053	Luteolin	-8.1
	20474	3'-Hydroxymelanettin	-8.1
	20345	Isodalbergin	-8.1
	4652	4-methylenemiltirone	-8.1
	14649	Methyl tanshinonate	-8.1
	32460	Butin	-8
	4530	miltionone I	-8
	20477	Dalbergin	-7.9
	32358	Apigenin	-7.9
	18414	Physcion	-7.8
	6256	Danshenxinkun A	-7.8
	4647	Saprothoquinone	-7.8
	18459	Jaranol	-7.8
	28203	salviolone	-7.8
	20476	Stevein	-7.8
	9481	Danshenxinkun D	-7.7
	21076	Biochanin A	-7.7
	32369	Kaempferol	-7.7
	32364	Daidzein	-7.7
	18458	Rhamnocitrin	-7.7
	3624	Isoimperatorin	-7.6
	20991	Koparin	-7.6
	19365	3'-Methoxydaidzein	-7.6
	4649	Aethiopinone	-7.5
	32837	Salvigenin	-7.5
	20514	Isoparvifuran	-7.5
	20598	Bolusanthin IV	-7.5
	19382	Isorhamnetin	-7.5
	18522	Prunetin	-7.5
	19378	2-(4-hydroxy-3-methoxyphenyl)-5-(3-hydroxypropyl)-7-metho	
		xy-3-benzofurancarboxaldehyde	-7.4
	21929	Mono-O-methylwightin	-7.4
	20628	Odoriflavene	-7.3
	18349	2',7-Dihydroxy-4',5'-dimethoxyisoflavone	-7.2
	20988	Xenognosin B	-7.1
	29621	Daphneolone	-7.1
	16012	Dalbergenone	-7.1
	20847	Isomucronustyrene	-7
	32247	Phloretol	-6.9
	8881	Neotigogenin	-6.9
	32504	7,3',4'-Trihydroxyflavone	-6.8
	21511	Methylnissolin	-6.8
	16014	Bowdichione	-6.7
	18640	2'-O-Methyl isoliquiritigenin	-6.6
	8390	Samaderin A	-6.3
	5019	FITONE	-5.5
	15758	Bifendate	-5.1
	11537	hexadecane	-5
	11666	Oktadekan	-5
	28390	Pubescene A	-4.9
	11555	Ethylpalmitate	-4.9
	11487	Myristic acid	-4.8
	11423	tridecane	-4.6
	26864	Abrisapogenol D	-4.3
	11697	Stearic acid	NA
	11741	Docosanoic acid	NA
	11906	Octadecanol	NA
	11729	Heneicosane	NA
	11756	Pentacosane	NA
	11878	Henicosyl formate	NA
	4524	Miltionone I	NA
	5816	(+)-8,11,13-Abietatrien-12-ol	NA
		5,6-dihydroxy-7-isopropyl-1,1-dimethyl-2,3-dihydrophenanthre	
	5841	n-4-one	NA
	12892	Urushio III	NA
	17604	4',5',7-trimethyl-3-methoxyflavone	NA
	18366	1,7-Dihydroxy-3,9-dimethoxy pterocarpene	NA
	20994	7-hydroxy-3-(2-hydroxy-3,4-dimethoxy-phenyl)chromone	NA
	28387	1,2-Dihydrotanshiquinone	NA
		1-methyl-8,9-dihydro-7H-naphtho[5,6-g]benzofuran-6,10,11-tri	
	28389	one	NA
	28394	Tanshinaldehyde II	NA

### 3.7 Evaluation of rat myocardial I/R injury model

**FIGURE 5:**
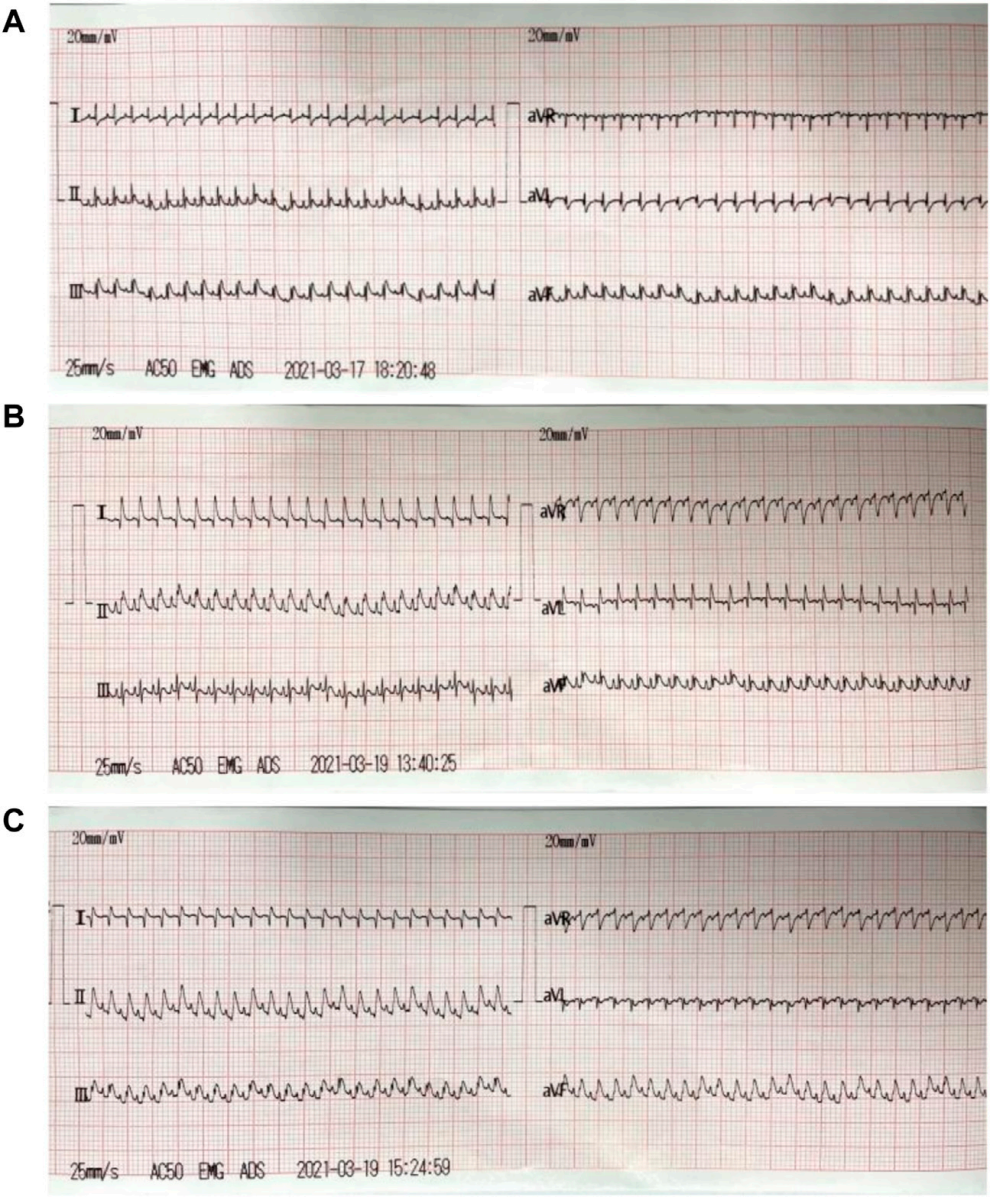
ECG of myocardial I/R injury model. **(A)**: ECG before modeling (the normal rats); **(B)**: ECG during acute myocardial ischemia: the acute myocardial ischemia was characterized by ST-segment elevation of multiple limb leads and high T wave, which was more significant of lead Ⅱ; **(C)**: ECG during reperfusion: the elevated ST-segment of lead Ⅱ returned to baseline level, and T wave returned to normal.

The ECG changes of rats were dynamically observed in real time by continuous monitoring of ECG limb lead. After ligation of left anterior descending coronary artery, the acute myocardial ischemia was characterized by ST-segment elevation of multiple limb leads and high T wave, which was more significant of lead Ⅱ. After 30 min of ischemia, the myocardium received reperfusion. The elevated ST-segment of lead Ⅱ returned to baseline level, and T wave returned to normal, which was accompanied by ventricular tachycardia, ventricular fibrillation and other arrhythmias (Figure 5).

### 3.8 Effect of Qishen Yiqi dripping pill on cardiac function

**FIGURE 6:**
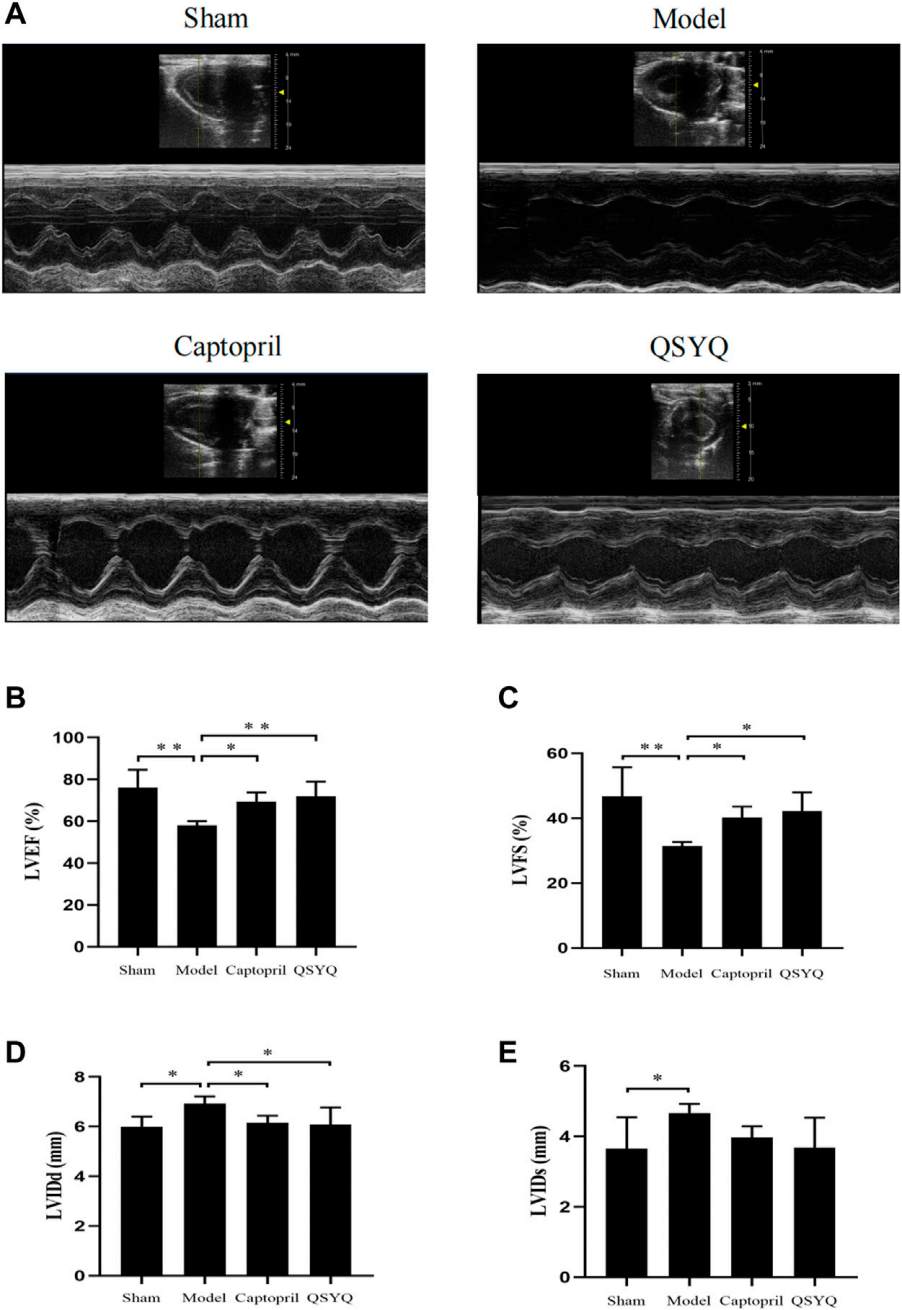
Effect of QSYQ on cardiac functional parameters. **(A)**: Representative M-mode images by echocardiography; **(B)**: Left ventricular ejection fraction, LVEF (%); **(C)**: Left ventricular fraction shortening, LVFS (%); **(D)**: Left ventricular end diastolic diameter, LVIDd (mm); **(E)**: Left ventricular end systolic diameter, LVIDs (mm), *n* = 4 per group. Data are expressed as mean ± SD. *p < 0.05, **p < 0.01.

The echocardiography results showed that compared with the sham group, the LVEF and LVFS were significantly decreased (*p* < 0.01), and the LVIDd and LVIDs were increased (*p* < 0.05) in model group. Compared with the model group, the LVEF and LVFS were increased (*p* < 0.05), and LVIDd was decreased (*p* < 0.05) in captopril group; the LVEF (*p* < 0.01) and LVFS (*p* < 0.05) were significantly increased, and LVIDd was decreased (*p* < 0.05) in QSYQ group. However, there was no significant change of LVIDs (*p* > 0.05) in both of the treatment groups (Figure 6).

### 3.9 Effect of Qishen Yiqi dripping pill on myocardial tissue pathological morphology

**FIGURE 7:**
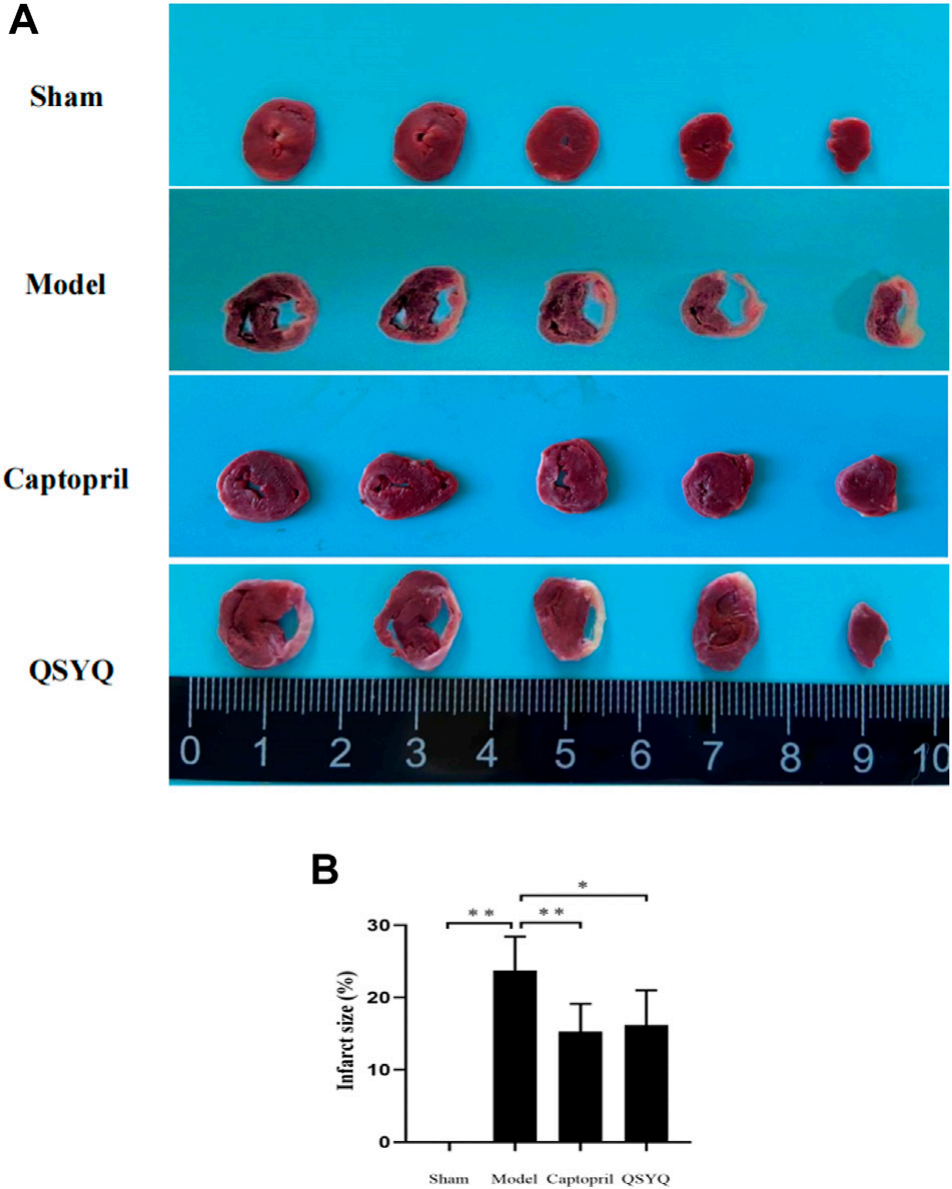
Effect of QSYQ on myocardial infarct size. **(A)**: TTC staining showed: in sham group, the myocardial tissues were with regular shape and ventricular enlargement was not observed. The model group showed morphological changes of myocardium, with the characteristic of left ventricular enlargement, and infarct ventricle walls turning thin and pale. The infarct size significantly increased in model group. Compared with the model group, the infarct size significantly decreased in both the captopril and QSYQ groups. **(B)**: quantitative analysis of QSYQ on myocardial infarct size, *n* = 4 per group. Data are expressed as mean ± SD. *p < 0.05, **p < 0.01.

TTC staining showed that in sham group, the myocardial tissues were with regular shape, good elasticity, and stained red. Ventricular enlargement was not observed. Compared with the sham group, the model group showed morphological changes of myocardium, with the characteristic of left ventricular enlargement, and infarct ventricle walls turning thin and pale. The infarct size significantly increased in model group (*p* < 0.01). Compared with the model group, the infarct size significantly decreased in both the captopril (*p* < 0.01) and QSYQ (*p* < 0.05) groups. There was no significant difference of the infarct size (*p* > 0.05) between the two treatment groups (Figure 7).

**FIGURE 8:**
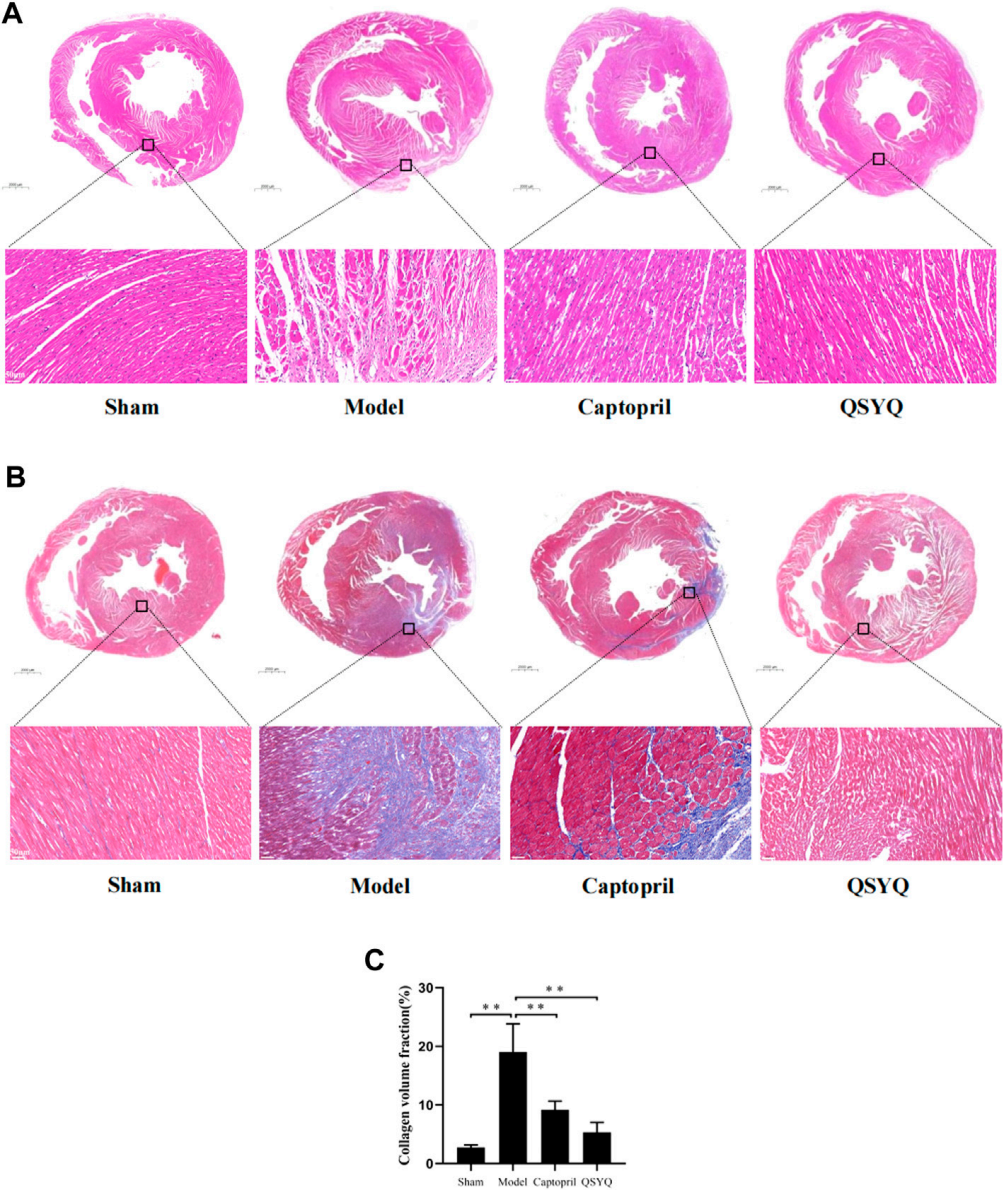
Effect of QSYQ on pathological morphology and collagen deposition of myocardium. **(A)**: HE staining showed that in model group myocardial fibers were disordered, and appeared swelling, fracture, degeneration and necrosis, accompanied by extensive inflammatory infiltration. Compared with model group, the morphology of myocardial tissue was improved in both the captopril and QSYQ groups. The myocardial fibers were well arranged, and swelling and inflammatory infiltration of myocardial interstitium were significantly alleviated, *n* = 4 per group. **(B)**: Masson staining of myocardial tissue showed that in model group the myocardial fibers arranged disorderly. There was a large amount of blue-stained collagen fiber deposition in the myocardial interstitial, and the fibrosis was more obvious at the edge of infarction. The CVF was significantly increased in model group. Compared with the model group, the CVF was decreased in both of the captopril and QSYQ groups. **(C)**: Quantitative analysis of QSYQ on CVF, *n* = 4 per group. Data are expressed as mean ± SD. **p < 0.01.

HE staining of myocardial tissue showed that in sham group, the myocardial fibers were orderly arranged and uniformly colored. There was no swelling, fracture, degeneration and necrosis of myocardial fibers. There was no inflammatory infiltration of myocardial interstitium. Compared with the sham group, the model group showed that cardiomyocytes were irregular in shape with large and hyperchromatic nuclei. The myocardial fibers were disordered, and appeared swelling, fracture, degeneration and necrosis, accompanied by extensive inflammatory infiltration. Compared with the model group, the morphology of myocardial tissue was improved in both the captopril and QSYQ groups. The myocardial fibers were well arranged. The myocardial fibers swelling and inflammatory infiltration of myocardial interstitium were significantly alleviated (Figure 8A).

Masson staining of myocardial tissue showed that in sham group the myocardial fibers arranged orderly. There were a small amount of blue-stained collagen fibers in the myocardial tissue. The collagen fibers were mainly distributed around blood vessels, and there were no obvious collagen fibers in the myocardial interstitium. Compared with the sham group, the model group showed that the myocardial fibers arranged disorderly. There was a large amount of blue-stained collagen fiber deposition in the myocardial interstitial, and the fibrosis was more obvious at the edge of infarction. The CVF was significantly increased in model group (*p* < 0.01). Compared with the model group, the CVF was decreased in both of the captopril and QSYQ groups (*p* < 0.01). It indicated that captopril and QSYQ could reduce the collagen deposition of myocardium. However, there was no significant difference of CVF (*p* > 0.05) in the two treatment groups (Figures 8B,C).

**FIGURE 9:**
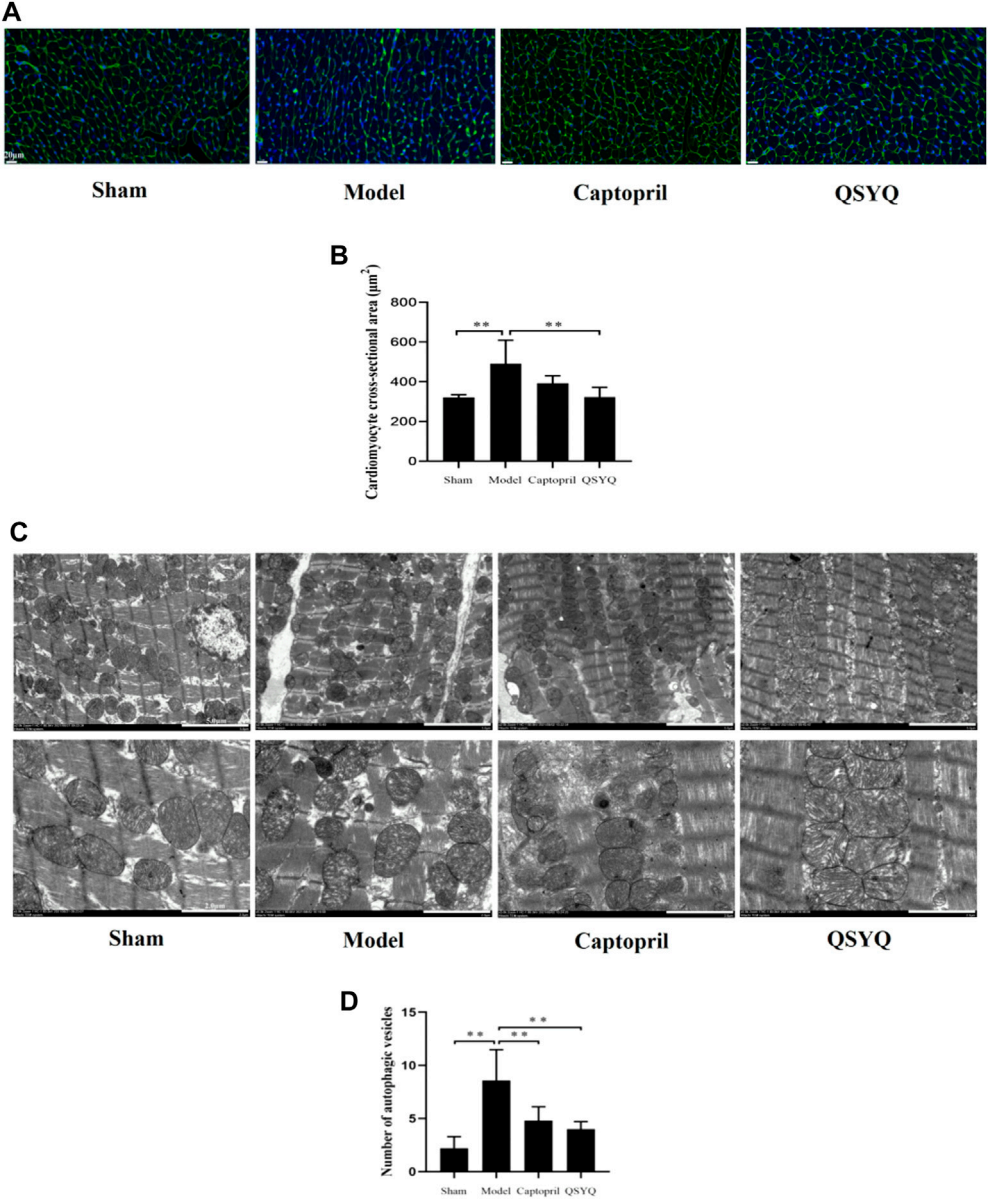
Effect of QSYQ on cardiomyocyte cross-sectional area and myocardial autophagosomes. **(A)**: WGA staining of myocardial tissue showed that in model group the myocardial fibers were disordered, the number of cardiomyocytes decreased, and the cross-sectional area of cardiomyocytes significantly increased. Compared with the model group, the outline of cardiomyocytes was regular, and the cross-sectional area of cardiomyocytes was significantly decreased in QSYQ group. **(B)**: quantitative analysis of QSYQ on cardiomyocyte cross-sectional area, *n* = 4 per group. Data are expressed as mean ± SD. **p < 0.01. **(C)**: in model group the myocardium ultrastructure was damaged. The myocardial membrane was damaged, the myocardial cell appeared edema, and the myofilaments were dissolved and partially ruptured. The number of autophagic vesicles increased in model group. The ultrastructure of myocardium showed attenuated injury in both of the captopril and QSYQ groups. The number of autophagic vesicles decreased in both of the treatment groups. **(D)**: quantitative analysis of QSYQ on myocardial autophagosomes, *n* = 5 per group. Data are expressed as mean ± SD. **p < 0.01.

WGA staining of myocardial tissue showed the outline of cardiomyocytes stained green fluorescence, and the nucleus stained blue fluorescence. In sham group, the outline of cardiomyocytes was regular and the myocardial fibers arranged orderly. Compared with the sham group, the model group showed that the myocardial fibers were disordered, the number of cardiomyocytes decreased, and the cross-sectional area of cardiomyocytes significantly increased (*p* < 0.01). Compared with the model group, the outline of cardiomyocytes was regular, and the cross-sectional area of cardiomyocytes was significantly decreased in QSYQ group (*p* < 0.01); however, there was no significant change of cardiomyocyte cross-sectional area in captopril group (*p* > 0.05) (Figures 9A,B).

### 3.10 Effect of Qishen Yiqi dripping pill on myocardial autophagosomes and myocardium ultrastructure

In the sham group the myocardium ultrastructure was normal. The myocardial membrane was intact, the myofilaments were neatly arranged, and the mitochondria retained clear and integrated structure with compact cristae. There were a small number of autophagic vesicles in myocardial tissues. In the model group the myocardium ultrastructure was damaged. The myocardial membrane was damaged, the myocardial cell appeared edema, and the myofilaments were dissolved and partially ruptured. Additionally, the mitochondria were swelled and ruptured with lysed cristae. Compared with the sham group the number of autophagic vesicles increased in model group (*p* < 0.01). Compared with the model group the ultrastructure of myocardium. showed attenuated injury in both of the captopril and QSYQ groups. The number of autophagic vesicles decreased in both of the treatment groups (*p* < 0.01) (Figures 9C,D).

### 3.11 Effect of Qishen Yiqi dripping pill on myocardial autophagy-related proteins and PI3K/Akt-mTOR signaling pathway

**FIGURE 10:**
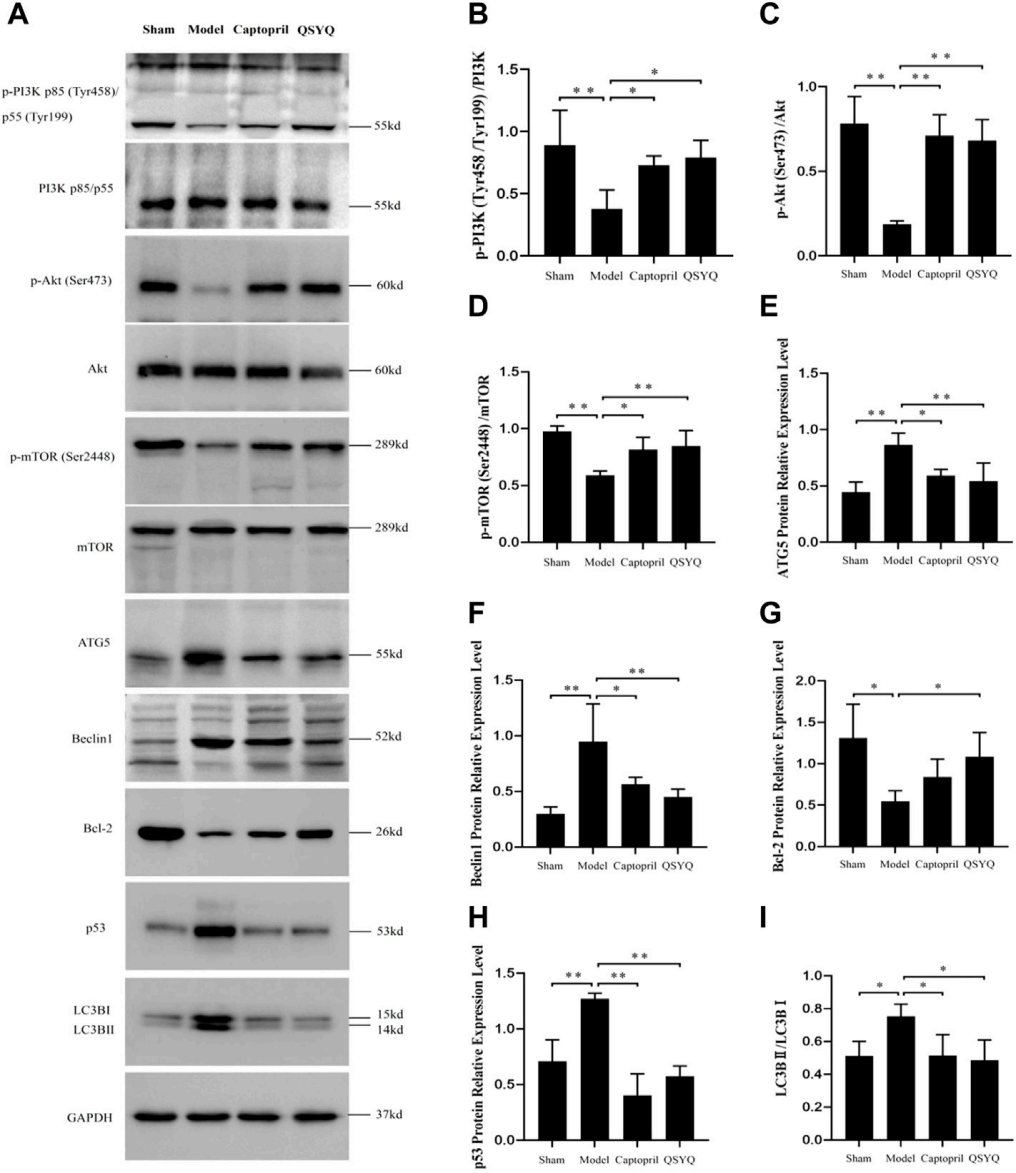
Effect of QSYQ on myocardial autophagy-related proteins and PI3K/Akt-mTOR signaling pathway. **(A)**: Detect the effect of QSYQ on myocardial autophagy-related proteins and PI3K/Akt-mTOR signaling pathway by Western blot in each group. **(B–I)**: Semi-quantitative analysis of the results, *n* = 3 per group. *p < 0.05, **p < 0.01.

**FIGURE 11:**
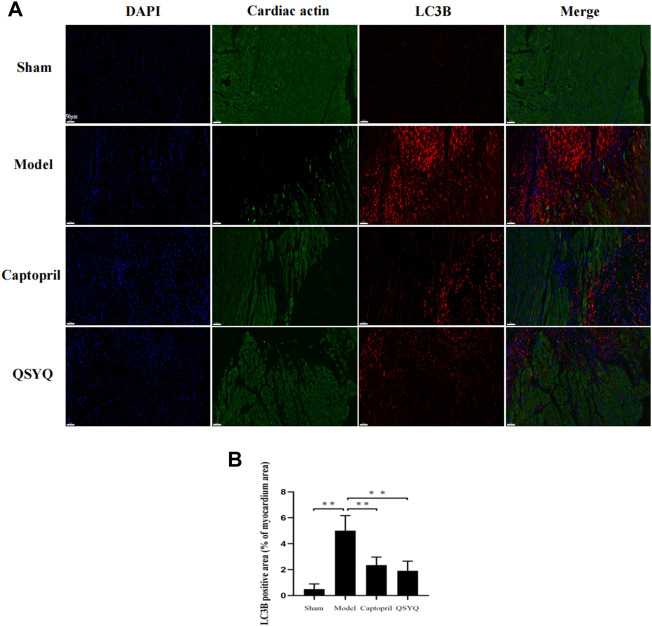
Effect of QSYQ on myocardial autophagy-related proteins. **(A)**: immunofluorescence staining showed: LC3B significantly increased in model group. After treatment with captopril or QSYQ, the expression of LC3B significantly decreased (Magnification, × 200). **(B)**: quantitative analysis of LC3B expression in myocardium, *n* = 4 per group. Data are expressed as mean ± SD. **p < 0.01.

Western blot showed the p-PI3K (Tyr458/Tyr199), p-Akt (Ser473), and p-mTOR (Ser2448) proteins expression were downregulated in the model group (*p* < 0.01), which indicated the PI3K/Akt-mTOR signaling pathway was inhibited. Autophagy-related proteins were detected, ATG5, Beclin1, p53, and LC3BⅡ/Ⅰ significantly increased (*p* < 0.01 or *p* < 0.05); Bcl-2 protein expression decreased in the model group (*p* < 0.05). The results indicated that autophagy was excessively activated and probably by inhibiting the PI3K/Akt-mTOR signaling pathway in model group. After QSYQ intervention, the excessive autophagy was restrained, and the PI3K/Akt-mTOR signaling pathway was activated. The QSYQ and captopril had similar effects on excessive autophagy in myocardial I/R injury model (Figure 10). To further confirm, we detected the LC3B by immunofluorescence staining. The result showed LC3B significantly increased in model group (*p* < 0.01). After treatment with captopril or QSYQ, the expression of LC3B significantly decreased (*p* < 0.01) (Figure 11).

### 3.12 Effect of Qishen Yiqi dripping pill on NLRP3 inflammasome

**FIGURE 12:**
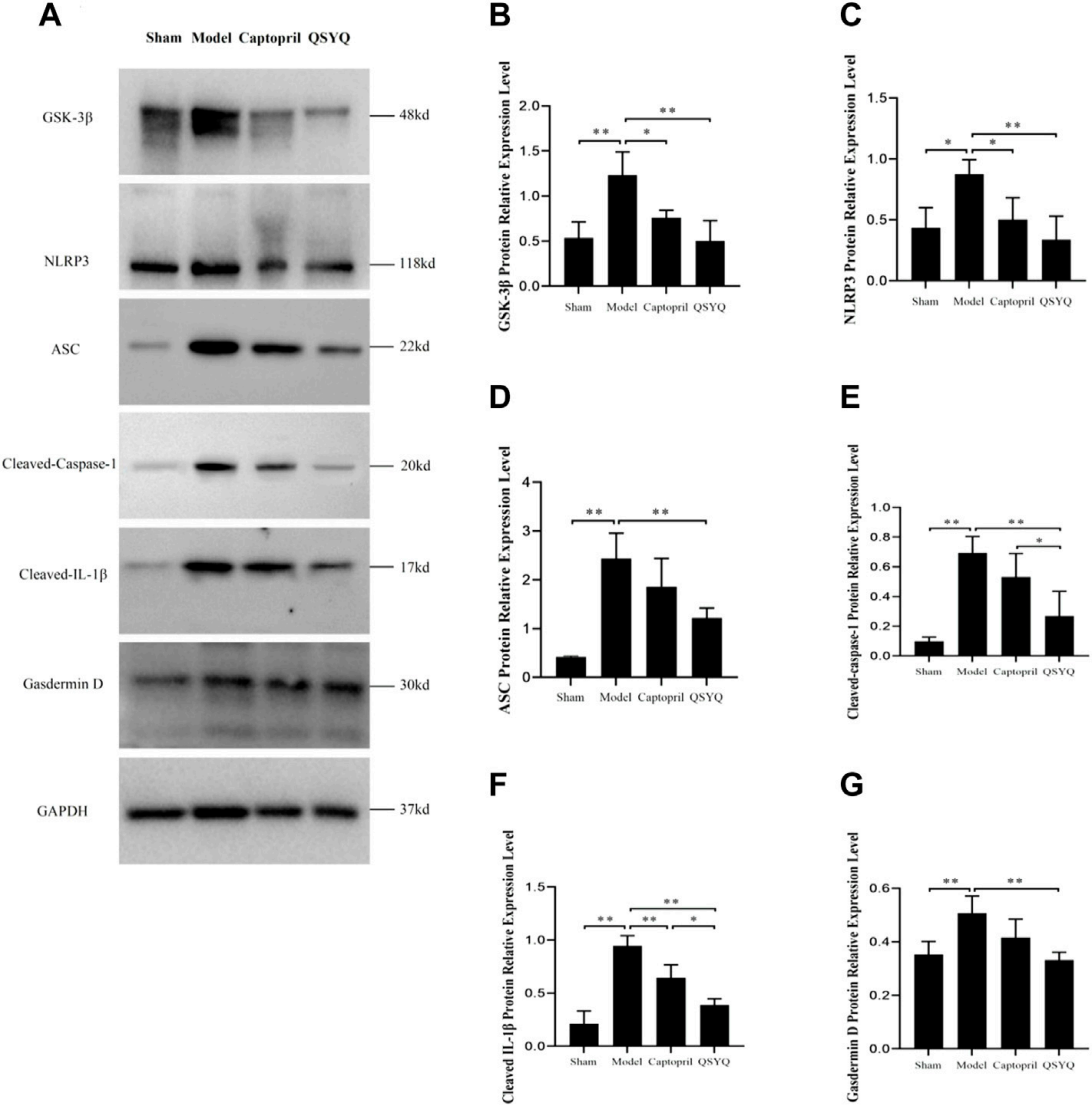
Effect of QSYQ on NLRP3 Inflammasome. **(A)**: Detect the effect of QSYQ on NLRP3 Inflammasome by Western blot in each group. **(B–G)**: Semi-quantitative analysis of the results, *n* = 3 per group. *p < 0.05, **p < 0.01.

**FIGURE 13:**
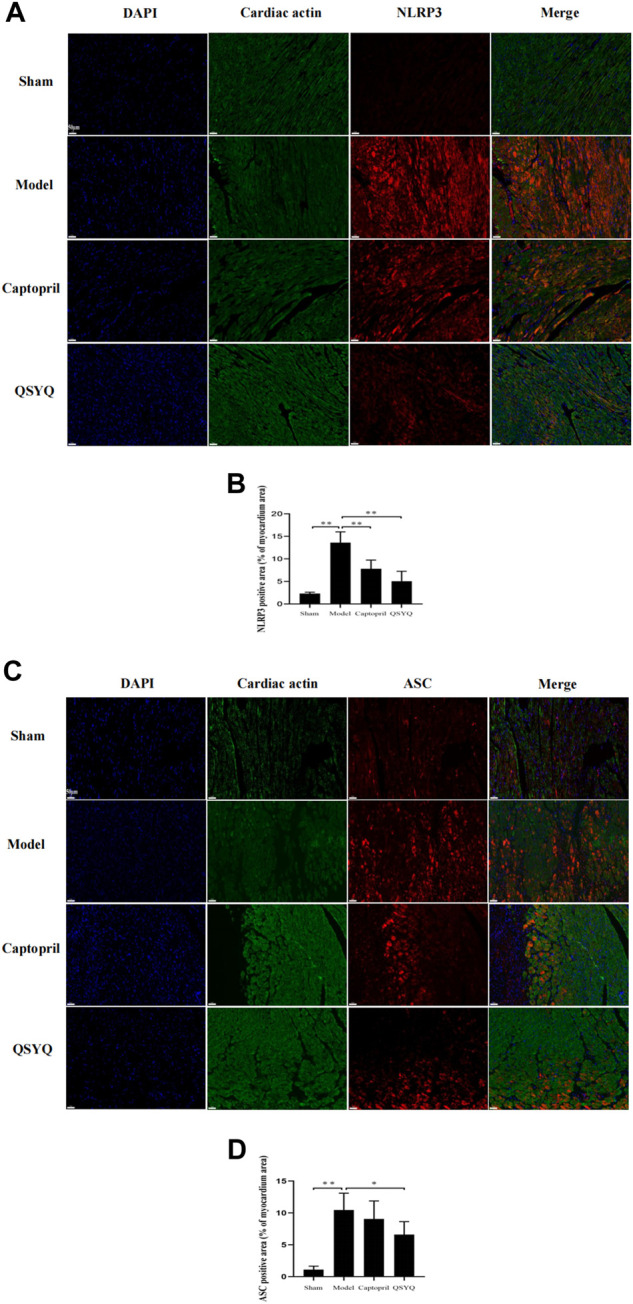
Effect of QSYQ on NLRP3 and ASC expression. **(A)**: Immunofluorescence staining showed: the expression of NLRP3 was upregulated in model group. After treatment with captopril or QSYQ, the NLRP3 expression significantly decreased in both of the treatment groups (Magnification, × 200). **(B)**: Quantitative analysis of NLRP3 expression in myocardium, *n* = 4 per group. Data are expressed as mean ± SD. **p < 0.01. **(C)**: Immunofluorescence staining showed: the expression of ASC was up-regulated in model group. The ASC expression decreased in QSYQ group, however, there was no significant change in captopril group (Magnification, × 200). **(D)**: Quantitative analysis of ASC expression in myocardium, *n* = 4 per group. Data are expressed as mean ± SD. *p < 0.05, **p < 0.01.

**FIGURE 14:**
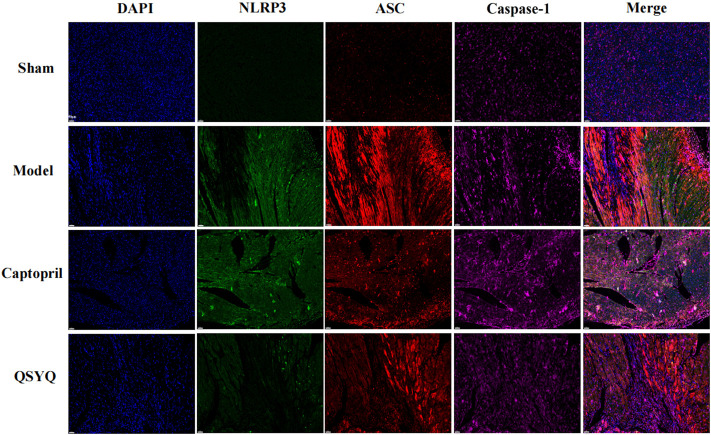
Effect of QSYQ on NLRP3 inflammasome assembly. Immunofluorescence staining was used to observe the co-localization of NLRP3, ASC and Caspase-1 in myocardium. The results showed the co-localization of NLRP3, ASC and Caspase-1 in model group, and the co-localization of NLRP3, ASC and Caspase-1 was significantly reduced with QSYQ treatment (NLRP3, green; ASC, yellow red; Caspase-1, red; DAPI, blue; Magnification, × 200; *n* = 4 per group).

Western blot showed GSK-3β, NLRP3, ASC, Cleaved-Caspase-1, Cleaved-IL-1β, and Gasdermin D proteins expression were increased in the model group (*p* < 0.01 or *p* < 0.05), which indicated NLRP3 inflammasome activation and pyroptosis progression. After QSYQ intervention, the GSK-3β, NLRP3, ASC, Cleaved-Caspase1, Cleaved-IL-1β, and Gasdermin D proteins expression were significantly decreased (*p* < 0.01), suggesting inhibition of NLRP3 inflammasome and pyroptosis. However, captopril could only downregulated GSK-3β (*p* < 0.05), NLRP3 (*p* < 0.05) and Cleaved-IL-1β expression (*p* < 0.01), and had no significant effect on other pyroptosis-related proteins (*p* > 0.05) (Figure 12). Immunofluorescence staining was used to further verify that compared with the sham group, the expression of NLRP3 and ASC were up-regulated in model group (*p* < 0.01, Figures 13A,B). After treatment with captopril or QSYQ, the NLRP3 expression significantly decreased in both of the treatment groups (*p* < 0.01, Figures 13A,B). The ASC expression decreased in QSYQ group (*p* < 0.05), however there was no significant change in captopril group (*p* > 0.05, Figures 13C,D). In addition, we investigated whether NLRP3 inflammasomes were assembled by immunofluorescence staining, and we observed the co-localization of NLRP3, ASC and Caspase-1 in model group. In QSYQ group, the co-localization of NLRP3, ASC and Caspase-1 was significantly reduced (Figure 14).

## 4 Discussion

Myocardial I/R injury is associated with serious clinical manifestations, which causes high rates of cardiac dysfunction and heart failure. However, currently there is no effective treatment to prevent this damage. In this study, the “bioactive compound-target-disease network” was constructed by network pharmacology to explore the underlying mechanism of QSYQ intervention on myocardial I/R injury, and the binding association between target genes and pivotal active ingredients was verified by molecular docking technology. Furthermore, we confirmed it *in vivo* that QSYQ had preventive and protective effects on myocardial I/R injury, potentially by inhibiting excessive autophagy in a PI3K/Akt/mTOR-dependent manner and suppressing NLRP3 inflammasome mediated pyroptosis.

Under normal conditions, autophagy occurs at a basal level of the heart and maintains cellular homeostasis by removing senescent organelles and excessive or long-lived proteins. Since the regenerative capacity of cardiomyocytes is very limited after mitosis, autophagy seems to be essential for maintaining normal structure and function of cardiomyocytes (Sciarretta et al., 2018a). Therefore, study that investigated the complex roles of autophagy in myocardial I/R injury is of particular importance. Accumulating evidence suggests that autophagy is upregulated in a short period of ischemia and further enhanced during reperfusion phase, which can be a “double-edged sword” in the pathological process of myocardial I/R injury. During ischemia/hypoxia phase, nutrient depletion induces autophagy to replenish metabolic substrates and remove damaged organelles, which provides at least a temporary reprieve for the threatened myocardium. Therefore, in ischemia/hypoxia phase, autophagy is an adaptive response with cardioprotective effects (Wang et al., 2018). The mechanism of autophagy in reperfusion phase is different from that in ischemia phase. During reperfusion phase, oxidative stress, calcium overload, endoplasmic reticulum stress and mitochondrial dysfunction promote uncontrolled excessive autophagy. Excessive autophagy can delete necessary proteins and organelles, leading to autophagic cardiomyocyte death and further aggravating myocardial injury (Dong et al., 2019). However, a new interpretation has recently emerged that autophagy flux is partly impaired in reperfusion rather than a single enhanced autophagy. In addition, when the autophagosomes cannot fuse with lysosomes and digest their contents, cardiomyocytes may eject the autophagosomes as a response, inducing an acute and intense inflammatory reaction (Yao et al., 2019).

The PI3K/Akt-mTOR signal pathway has an essential cardioprotective effect, which is particularly important for promoting cardiomyocyte survival against myocardial I/R injury (Li et al., 2018). mTOR plays an essential role in regulating cell growth and metabolism in response to energy stress, nutrients and growth factors. Moreover, mTOR is a crucial regulator of autophagy. There are two types of mTOR complexes. mTOR complex 1 (mTORC1) consists of mTOR, proline-rich AKT1 substrate 40 (PRAS40), and regulatory associated protein of mTOR (Raptor). mTORC1 plays a vital role in cell growth by regulating transcription, translation and autophagy. Ras homolog enriched in brain (Rheb) and tuberous sclerosis complex (TSC)1/TSC2 are the most critical regulators of mTORC1 activity. Akt can activate mTORC1 in different ways (Sciarretta et al., 2018b; Shi et al., 2019). Akt inhibits the assembly of TCS1/TCS2, which relieves the inhibition of Rheb on mTORC1; for another, Akt phosphorylates TSC2 and inhibits its GAP domain, leading to activation of Rheb and mTORC1; furthermore, Akt/PKB induces phosphorylation of PRAS40 and eliminates its inhibiting effect on mTORC1 (Mallela and Kumar, 2021; Qin et al., 2021).

Beclin1, the mammalian homologue of yeast Atg6, plays an essential role in regulating autophagosome formation and processing, especially in reperfusion phase (Xu and Qin, 2019). Accumulating evidence indicates Beclin1 overexpression enhanced autophagy activity during I/R *in vitro*; conversely, depletion of Beclin1 by siRNA or Beclin1 mutation mice reduced autophagy during reperfusion (Hamacher-Brady et al., 2006; Valentim et al., 2006; Tsai et al., 2021). Under I/R condition, excessive autophagy is induced by the class III PI3K complex, which contains class Ш PI3K Vps34 and Beclin1. The coiled-coil domain of Beclin1 can bind to class III PI3K Vps34 by interacting with an evolutionarily conserved domain (Zhao et al., 2020). Recently, mounting studies have found autophagy is inhibited when Bcl-2 binds to Beclin1. A study has reported that ischemic preconditioning (IPC) attenuated Beclin1-dependent excessive autophagy by inhibiting Bcl-2 dissociation from Beclin1 in reperfusion phase, which protected the rat heart against I/R injury (Peng et al., 2013). Bcl-2 can also inhibit Beclin1/class III PI3K Vps34 complex formation and the activity of Beclin1-associated class III PI3K. In addition, the class III PI3K autophagic pathway is inhibited by Bcl-2 binding to Beclin1 (Wang et al., 2020a). p53 is an important regulator of autophagy and apoptosis (White, 2016). The study has reported that p53 upregulation played a significant role in contractile dysfunction during the late phase of reperfusion. However, p53 did not participate in cardiomyocyte necrosis during ischemia or early stage of reperfusion (Yano et al., 2018).

The significance of NLRP3 inflammasome in myocardial I/R injury has become a hotspot of current research. Increasing evidence suggests NLRP3 is an initial receptor for inflammasome activation and myocardial I/R injury. Assembly of NLRP3 inflammasome induces the release of caspase-1 dependent pro-inflammatory cytokines IL-1β and IL-18, as well as gasdermin D mediated pyroptosis. Researchers reported that after myocardial infarction, NLRP3 inflammasome components were primarily upregulated in left ventricle and mainly distributed in myocardial fibroblasts. Myocardial fibroblasts have been shown to stimulate IL-1β and IL-18 release *in vitro*, and played an important role in inflammasome activation (Kawaguchi et al., 2011; Mezzaroma et al., 2011). Additionally, it was reported that hearts isolated from NLRP3 deficient mice were perfused and subjected to global ischemia and reperfusion, which showed significant improvement in cardiac function and remission of hypoxia damage compared with wild-type hearts (Sandanger et al., 2013). GSK-3β is a serine/threonine kinase that participates in signaling pathways via phosphorylation-mediated signal cascades, and cell survival is largely dependent on it. GSK-3β also acts as a molecular determinant of the spatio-temporal dynamics of NLRP3 inflammasome activation. Researches have shown that GSK-3β aggravated cardiac hypertrophy (Matsuda et al., 2008) and heart failure (Hirotani et al., 2007). Furthermore, GSK-3β inhibition alleviated myocardial ischemia reperfusion and doxorubicin induced heart damage (Miura and Tanno, 2010). A recent study showed that GSK-3β inhibition alleviated NLRP3 inflammasome activation in myocardial infarction, and subsequently affected IL-1β release (Wang et al., 2020b). However, some viewpoints remain controversial. Recently, some evidence suggested that NLRP3 inflammation might be a double-edged sword (Zuurbier, 2019). NLRP3 inflammation is detrimental when overactivated, but beneficial when moderately activated, dependenting on reperfusion time and cellular location. Indeed, several other components of the inflammatory response system, such as low-level stimulation of TNFa (Lecour et al., 2002), IL-6 (Zuurbier et al., 2012), toll-like receptor 2 (TLR2) (Dong et al., 2010), and home-mobility group box 1 (HMGB1) (Kitahara et al., 2008) have been detected to protect against I/R injury. The double-edged effect of NLRP3 inflammation remains further study.

The present study explored whether QSYQ had effect on alleviating myocardial I/R injury and improving cardiac function and structure by regulating autophagy and NLRP3 Inflammasome. The results showed QSYQ could significantly improve cardiac function by increasing LVEF and LVFS, and decreasing LVIDd, and its effect was not significantly different from captopril. Histopathological observation showed QSYQ and captopril had effect on improving myocardial pathological morphology. Both of the QSYQ and captopril could reduce myocardial infarction size, alleviate myocardial fibers swelling and inflammatory infiltration, and decrease collagen fiber deposition. QSYQ could also relieve cardiomyocyte hypertrophy, however, captopril could not improve cardiomyocyte hypertrophy. It is noteworthy that reparative myocardial fibrosis caused by cardiomyocyte necrosis could develop scars by replacing small lesions of cardiomyocyte necrosis. Reactive myocardial fibrosis triggered by ischemic injury could expand and thicken the myocardial muscle bundles and its surrounding fibrous tissue, leading to abnormal myocardial structure and cardiac systolic dysfunction. Therefore, effective control of myocardial fibrosis is essential for prevention and treatment of myocardial I/R injury. Further research observed the myocardial autophagosomes and myocardium ultrastructure by transmission electron microscope, and found QSYQ decreased the number of autophagic vesicles and attenuated myocardium ultrastructure injury. The mechanism study found QSYQ could regulate autophagy-related proteins and activate PI3K/Akt-mTOR signaling pathway, consequently inhibited the excessive autophagy under I/R injury; furthermore, QSYQ could inhibit the activation and assembly of NLRP3 inflammasome, and thus suppressed pyroptosis.

In conclusion, QSYQ effectively alleviated myocardial I/R injury and improved cardiac function and structure, possibly by regulating autophagy-related proteins and PI3K/Akt-mTOR signaling pathway, thus inhibiting the excessive autophagy. In addition, QSYQ significantly inhibited the activation and assembly of NLRP3 Inflammasome, and thus suppressed pyroptosis.

However, there are some limitations to the present study. Firstly, this study only observed the excessive autophagy in myocardial I/R injury. Nevertheless, whether the autophagy flux is partly impaired and if QSYQ has effect on it remain unknown. Secondly, this study did not further explore the dynamic changes of autophagy process during ischemia and reperfusion phase, and whether QSYQ has different effects on it also needs further research.

## Data Availability

The original contributions presented in the study are included in the article/Supplementary Material, further inquiries can be directed to the corresponding authors.

## Supplementary material

Supplementary Table S1 Metabolite and potential targets of QSYQ. After the preliminary pharmacokinetic screening, a total of 111 metabolite components (20 in Huangqi, 65 in Danshen, 1 in Sanqi , and 33 in Jiangxiang), and 477 targets were obtained.

Supplementary Table S2 Myocardial I/R injury-related targets. By searching the TTD and DisgenetGene databases and deleting duplicate values, a total of 178 myocardial I/R injury-related targets were identified.

## References

[B1] Aghaei M. Motallebnezhad M. Ghorghanlu S. Jabbari A. Enayati A. Rajaei M. (2019). Targeting autophagy in cardiac ischemia/reperfusion injury: A novel therapeutic strategy. J. Cell. Physiol. 234 (10), 16768–16778. 10.1002/jcp.28345 30807647

[B2] Arumugam S. Qin Y. Liang Z. Han S. N. Boodapati S. L. T. Li J. (2022). GSK3β mediates the spatiotemporal dynamics of NLRP3 inflammasome activation. Cell. Death Differ. 10.1038/s41418-022-00997-y PMC9525599 35477991

[B3] Cao W. Li J. Yang K. Cao D. (2021). An overview of autophagy: Mechanism, regulation and research progress. Bull. Cancer 108 (3), 304–322. 10.1016/j.bulcan.2020.11.004 33423775

[B4] Chang M. Cheng L. Shen Y. Zhang Y. Zhang Z. Hao P. (2019). Qishenyiqi dripping pill improves ventricular remodeling and function in patients with chronic heart failure: A pooled analysis. Med. Baltim. 98 (2), e13906. 10.1097/MD.0000000000013906 PMC6336621 30633164

[B5] Chen J. R. Wei J. Wang L. Y. Zhu Y. Li L. Olunga M. A. (2015). Cardioprotection against ischemia/reperfusion injury by QiShenYiQi Pill® via ameliorate of multiple mitochondrial dysfunctions. Drug Des. devel. Ther. 9, 3051–3066. 10.2147/DDDT.S82146 PMC4474392 26109848

[B6] Cheng J. Zhang W. Zhang X. Han F. Li X. He X. (2014). Effect of angiotensin-converting enzyme inhibitors and angiotensin II receptor blockers on all-cause mortality, cardiovascular deaths, and cardiovascular events in patients with diabetes mellitus: A meta-analysis. JAMA Intern. Med. 174 (5), 773–785. 10.1001/jamainternmed.2014.348 24687000

[B7] Dong J. W. Vallejo J. G. Tzeng H. P. Thomas J. A. Mann D. L. (2010). Innate immunity mediates myocardial preconditioning through Toll-like receptor 2 and TIRAP-dependent signaling pathways. Am. J. Physiol. Heart Circ. Physiol. 298 (3), H1079–H1087. 10.1152/ajpheart.00306.2009 20061547 PMC2838552

[B8] Dong Y. Chen H. Gao J. Liu Y. Li J. Wang J. (2019). Molecular machinery and interplay of apoptosis and autophagy in coronary heart disease. J. Mol. Cell. Cardiol. 136, 27–41. 10.1016/j.yjmcc.2019.09.001 31505198

[B9] Ferrari R. Rosano G. M. (2013). Not just numbers, but years of science: Putting the ACE inhibitor-ARB meta-analyses into context. Int. J. Cardiol. 166 (2), 286–288. 10.1016/j.ijcard.2013.01.027 23452882

[B10] Guo Z. Yu S. Chen X. Ye R. Zhu W. Liu X. (2016). NLRP3 is involved in ischemia/reperfusion injury. CNS Neurol. Disord. Drug Targets 15 (6), 699–712. 10.2174/1871527315666160321111829 26996163

[B11] Hamacher-Brady A. Brady N. R. Gottlieb R. A. (2006). Enhancing macroautophagy protects against ischemia/reperfusion injury in cardiac myocytes. J. Biol. Chem. 281 (40), 29776–29787. 10.1074/jbc.M603783200 16882669

[B12] Han J. Y. Li Q. Pan C. S. Sun K. Fan J. Y. (2019). Effects and mechanisms of QiShenYiQi pills and major ingredients on myocardial microcirculatory disturbance, cardiac injury and fibrosis induced by ischemia-reperfusion. Pharmacol. Res. 147, 104386. 10.1016/j.phrs.2019.104386 31377222

[B13] He G. X. Xie J. Jiang H. Tan W. Xu B. (2018). Effects of qishen yiqi dripping pills in reducing myocardial injury and preserving microvascular function in patients undergoing elective percutaneous coronary intervention: A pilot randomized study. Chin. J. Integr. Med. 24 (3), 193–199. 10.1007/s11655-017-2955-1 28470563

[B14] He Y. Hara H. Núñez G. (2016). Mechanism and regulation of NLRP3 activation, inflammasome activation. Trends Biochem. Sci. 41 (12), 1012–1021. 10.1016/j.tibs.2016.09.002 27669650 PMC5123939

[B15] Heusch G. (2020). Myocardial ischaemia-reperfusion injury and cardioprotection in perspective. Nat. Rev. Cardiol. 17 (12), 773–789. 10.1038/s41569-020-0403-y 32620851

[B16] Hirotani S. Zhai P. Tomita H. Galeotti J. Marquez J. P. Gao S. (2007). Inhibition of glycogen synthase kinase 3beta during heart failure is protective. Circ. Res. 101 (11), 1164–1174. 10.1161/CIRCRESAHA.107.160614 17901358

[B17] Huang Y. Xu W. Zhou R. (2021). NLRP3 inflammasome activation and cell death. Cell. Mol. Immunol. 18 (9), 2114–2127. 10.1038/s41423-021-00740-6 34321623 PMC8429580

[B18] Huang Y. Zhang K. Liu M. Su J. Qin X. Wang X. (2021). An herbal preparation ameliorates heart failure with preserved ejection fraction by alleviating microvascular endothelial inflammation and activating NO-cGMP-PKG pathway. Phytomedicine. 91, 153633. 10.1016/j.phymed.2021.153633 34320423

[B19] Kawaguchi M. Takahashi M. Hata T. Kashima Y. Usui F. Morimoto H. (2011). Inflammasome activation of cardiac fibroblasts is essential for myocardial ischemia/reperfusion injury. Circulation 123 (6), 594–604. 10.1161/CIRCULATIONAHA.110.982777 21282498

[B20] Kitahara T. Takeishi Y. Harada M. Niizeki T. Suzuki S. Sasaki T. (2008). High-mobility group box 1 restores cardiac function after myocardial infarction in transgenic mice. Cardiovasc. Res. 80 (1), 40–46. 10.1093/cvr/cvn163 18558628

[B21] Lecour S. Smith R. M. Woodward B. Opie L. H. Rochette L. Sack M. N. (2002). Identification of a novel role for sphingolipid signaling in TNF alpha and ischemic preconditioning mediated cardioprotection. J. Mol. Cell. Cardiol. 34 (5), 509–518. 10.1006/jmcc.2002.1533 12056855

[B22] Li X. Hu X. Wang J. Xu W. Yi C. Ma R. (2018). Inhibition of autophagy via activation of PI3K/Akt/mTOR pathway contributes to the protection of hesperidin against myocardial ischemia/reperfusion injury. Int. J. Mol. Med. 42 (4), 1917–1924. 10.3892/ijmm.2018.3794 30066841 PMC6108872

[B23] Lin X. L. Xiao W. J. Xiao L. L. Liu M. H. (2018). Molecular mechanisms of autophagy in cardiac ischemia/reperfusion injury (Review). Mol. Med. Rep. 18 (1), 675–683. 10.3892/mmr.2018.9028 29845269

[B24] Lv S. Wang Q. Wu M. Li M. Wang X. Xu L. (2021). QiShenYiQi pill improves myocardial hypertrophy caused by pressure overload in rats. Evid. Based. Complement. Altern. Med. 2021, 5536723. 10.1155/2021/5536723 PMC8225423 34221074

[B25] Lv S. Yuan P. Dong J. Lu C. Li M. Qu F. (2020). QiShenYiQi pill improves the reparative myocardial fibrosis by regulating autophagy. J. Cell. Mol. Med. 24 (19), 11283–11293. 10.1111/jcmm.15695 32881330 PMC7576289

[B26] Mallela K. Kumar A. (2021). Role of TSC1 in physiology and diseases. Mol. Cell. Biochem. 476 (6), 2269–2282. 10.1007/s11010-021-04088-3 33575875

[B27] Matsuda T. Zhai P. Maejima Y. Hong C. Gao S. Tian B. (2008). Distinct roles of GSK-3alpha and GSK-3beta phosphorylation in the heart under pressure overload. Proc. Natl. Acad. Sci. U. S. A. 105 (52), 20900–20905. 10.1073/pnas.0808315106 19106302 PMC2634936

[B28] Mezzaroma E. Toldo S. Farkas D. Seropian I. M. Van Tassell B. W. Salloum F. N. (2011). The inflammasome promotes adverse cardiac remodeling following acute myocardial infarction in the mouse. Proc. Natl. Acad. Sci. U. S. A. 108 (49), 19725–19730. 10.1073/pnas.1108586108 22106299 PMC3241791

[B29] Miura T. Tanno M. (2010). Mitochondria and GSK-3beta in cardioprotection against ischemia/reperfusion injury. Cardiovasc. Drugs Ther. 24 (3), 255–263. 10.1007/s10557-010-6234-z 20490903

[B30] Mohr F. W. Morice M. C. Kappetein A. P. Feldman T. E. Ståhle E. Colombo A. (2013). Coronary artery bypass graft surgery versus percutaneous coronary intervention in patients with three-vessel disease and left main coronary disease: 5-year follow-up of the randomised, clinical SYNTAX trial. Lancet 381 (9867), 629–638. 10.1016/S0140-6736(13)60141-5 23439102

[B31] Mokhtari B. Badalzadeh R. (2021). The potentials of distinct functions of autophagy to be targeted for attenuation of myocardial ischemia/reperfusion injury in preclinical studies: An up-to-date review. J. Physiol. Biochem. 77, 377–404. 10.1007/s13105-021-00824-x 34173955

[B32] Peng W. Liu Y. Xu W. J. Xia Q. H. (2013). Role of Beclin 1-dependent autophagy in cardioprotection of ischemic preconditioning. J. Huazhong Univ. Sci. Technol. Med. Sci. 33 (1), 51–56. 10.1007/s11596-013-1070-6 23392707

[B33] Qin G. W. Lu P. Peng L. Jiang W. (2021). Ginsenoside Rb1 inhibits cardiomyocyte autophagy via PI3K/Akt/mTOR signaling pathway and reduces myocardial ischemia/reperfusion injury. Am. J. Chin. Med. 49 (8), 1913–1927. 10.1142/S0192415X21500907 34775933

[B34] Reed G. W. Rossi J. E. Cannon C. P. (2017). Acute myocardial infarction. Lancet 389 (10065), 197–210. 10.1016/S0140-6736(16)30677-8 27502078

[B35] Sandanger Ø. Ranheim T. Vinge L. E. Bliksøen M. Alfsnes K. Finsen A. V. (2013). The NLRP3 inflammasome is up-regulated in cardiac fibroblasts and mediates myocardial ischaemia-reperfusion injury. Cardiovasc. Res. 99 (1), 164–174. 10.1093/cvr/cvt091 23580606

[B36] Sciarretta S. Forte M. Frati G. Sadoshima J. (2018). New insights into the role of mTOR signaling in the cardiovascular system. Circ. Res. 122 (3), 489–505. 10.1161/CIRCRESAHA.117.311147 29420210 PMC6398933

[B37] Sciarretta S. Maejima Y. Zablocki D. Sadoshima J. (2018). The role of autophagy in the heart. Annu. Rev. Physiol. 80, 1–26. 10.1146/annurev-physiol-021317-121427 29068766

[B38] Shi B. Ma M. Zheng Y. Pan Y. Lin X. (2019). mTOR and Beclin1: Two key autophagy-related molecules and their roles in myocardial ischemia/reperfusion injury. J. Cell. Physiol. 234 (8), 12562–12568. 10.1002/jcp.28125 30618070 PMC13484135

[B39] Snow V. Barry P. Fihn S. D. Gibbons R. J. Owens D. K. Williams S. V. (2004). Primary care management of chronic stable angina and asymptomatic suspected or known coronary artery disease: A clinical practice guideline from the American college of physicians. Ann. Intern. Med. 141 (7), 562–567. 10.7326/0003-4819-141-7-200410050-00014 15466774

[B40] Song X. D. Feng J. P. Yang R. X. (2019). Alamandine protects rat from myocardial ischemia-reperfusion injury by activating JNK and inhibiting NF-κB. Eur. Rev. Med. Pharmacol. Sci. 23 (15), 6718–6726. 10.26355/eurrev_201908_18563 31378915

[B41] Tsai C. F. Su H. H. Chen K. M. Liao J. M. Yao Y. T. Chen Y. H. (2021). Paeonol protects against myocardial ischemia/reperfusion-induced injury by mediating apoptosis and autophagy crosstalk. Front. Pharmacol. 11, 586498. 10.3389/fphar.2020.586498 33551799 PMC7858273

[B42] Valentim L. Laurence K. M. Townsend P. A. Carroll C. J. Soond S. Scarabelli T. M. (2006). Urocortin inhibits Beclin1-mediated autophagic cell death in cardiac myocytes exposed to ischaemia/reperfusion injury. J. Mol. Cell. Cardiol. 40 (6), 846–852. 10.1016/j.yjmcc.2006.03.428 16697404

[B43] van-Vark L. C. Bertrand M. Akkerhuis K. M. Brugts J. J. Fox K. Mourad J. J. (2012). Angiotensin-converting enzyme inhibitors reduce mortality in hypertension: A meta-analysis of randomized clinical trials of renin-angiotensin-aldosterone system inhibitors involving 158, 998 patients. Eur. Heart J. 33 (16), 2088–2097. 10.1093/eurheartj/ehs075 22511654 PMC3418510

[B44] Wang S. Su X. Xu L. Chang C. Yao Y. Komal S. (2020). Glycogen synthase kinase-3β inhibition alleviates activation of the NLRP3 inflammasome in myocardial infarction. J. Mol. Cell. Cardiol. 149, 82–94. 10.1016/j.yjmcc.2020.09.009 32991876

[B45] Wang X. Guo Z. Ding Z. Mehta J. L. (2018). Inflammation, autophagy, and apoptosis after myocardial infarction. J. Am. Heart Assoc. 7 (9), e008024. 10.1161/JAHA.117.008024 29680826 PMC6015297

[B46] Wang X. Jiang Y. Zhu L. Cao L. Xu W. Rahman S. U. (2020). Autophagy protects PC12 cells against deoxynivalenol toxicity via the Class III PI3K/beclin 1/Bcl-2 pathway. J. Cell. Physiol. 235 (11), 7803–7815. 10.1002/jcp.29433 31930515

[B47] White E. (2016). Autophagy and p53. Cold Spring Harb. Perspect. Med. 6 (4), a026120. 10.1101/cshperspect.a026120 27037419 PMC4817743

[B48] Wu M. Y. Yiang G. T. Liao W. T. Tsai A. P. Cheng Y. L. Cheng P. W. (2018). Current mechanistic concepts in ischemia and reperfusion injury. Cell. Physiol. biochem. 46 (4), 1650–1667. 10.1159/000489241 29694958

[B49] Xu H. D. Qin Z. H. (2019). Beclin 1, bcl-2 and autophagy. Adv. Exp. Med. Biol. 1206, 109–126. 10.1007/978-981-15-0602-4_5 31776982

[B50] Xu S. Y. (2002). Pharmacological experimental methodology. Beijing: People's Health Publishing House, 200–223.

[B51] Yano T. Abe K. Tanno M. Miki T. Kuno A. Miura T. (2018). Does p53 inhibition suppress myocardial ischemia-reperfusion injury? J. Cardiovasc. Pharmacol. Ther. 23 (4), 350–357. 10.1177/1074248418763612 29554809 PMC6203944

[B52] Yao L. Chen H. Wu Q. Xie K. (2019). Hydrogen-rich saline alleviates inflammation and apoptosis in myocardial I/R injury via PINK-mediated autophagy. Int. J. Mol. Med. 44 (3), 1048–1062. 10.3892/ijmm.2019.4264 31524220 PMC6657957

[B53] Zhang Y. Shi P. Yao H. Shao Q. Fan X. (2012). Metabolite profiling and pharmacokinetics of herbal compounds following oral administration of a cardiovascular multi-herb medicine (Qishen yiqi pills) in rats. Curr. Drug Metab. 13, 510–523. 10.2174/1389200211209050510 22292791

[B54] Zhao Y. Zhu M. Chen W. Chang Q. Shen L. Yan R. (2020). TFPIα alleviated vascular endothelial cell injury by inhibiting autophagy and the class III PI3K/Beclin-1 pathway. Thromb. Res. 195, 151–157. 10.1016/j.thromres.2020.07.017 32702563

[B55] Zuurbier C. J. Jong W. M. Eerbeek O. Koeman A. Pulskens W. P. Butter L. M. (2012). Deletion of the innate immune NLRP3 receptor abolishes cardiac ischemic preconditioning and is associated with decreased Il-6/STAT3 signaling. PLoS One 7 (7), e40643. 10.1371/journal.pone.0040643 22848390 PMC3407219

[B56] Zuurbier C. J. (2019). NLRP3 inflammasome in cardioprotective signaling. J. Cardiovasc. Pharmacol. 74 (4), 271–275. 10.1097/FJC.0000000000000696 31356546

